# In-Depth Characterization of Somatic and Orofacial Sensitive Dysfunctions and Interfering-Symptoms in a Relapsing-Remitting Experimental Autoimmune Encephalomyelitis Mouse Model

**DOI:** 10.3389/fneur.2021.789432

**Published:** 2022-01-17

**Authors:** Amélie Démosthènes, Benoît Sion, Fabrice Giraudet, Xavier Moisset, Laurence Daulhac, Alain Eschalier, Mélina Bégou

**Affiliations:** ^1^Université Clermont Auvergne, Inserm, Neuro-Dol, Faculté de Pharmacie, Faculté de Médecine, Institut Analgesia, BP38, Clermont-Ferrand, France; ^2^Université Clermont Auvergne, CHU de Clermont-Ferrand, Inserm, Neuro-Dol, Faculté de Médecine, Institut Analgesia, BP38, Clermont-Ferrand, France

**Keywords:** sensitive dysfunctions, relapsing-remitting, EAE (experimental autoimmune encephalomyelitis), multiple sclerosis, mouse model

## Abstract

Among the many symptoms (motor, sensory, and cognitive) associated with multiple sclerosis (MS), chronic pain is a common disabling condition. In particular, neuropathic pain symptoms are very prevalent and debilitating, even in early stages of the disease. Unfortunately, chronic pain still lacks efficient therapeutic agents. Progress is needed (i) clinically by better characterizing pain symptoms in MS and understanding the underlying mechanisms, and (ii) preclinically by developing a more closely dedicated model to identify new therapeutic targets and evaluate new drugs. In this setting, new variants of experimental autoimmune encephalomyelitis (EAE) are currently developed in mice to exhibit less severe motor impairments, thereby avoiding confounding factors in assessing pain behaviors over the disease course. Among these, the optimized relapsing-remitting EAE (QuilA-EAE) mouse model, induced using myelin oligodendrocyte glycoprotein peptide fragment (35–55), pertussis toxin, and quillaja bark saponin, seems very promising. Our study sought (i) to better define sensitive dysfunctions and (ii) to extend behavioral characterization to interfering symptoms often associated with pain during MS, such as mood disturbances, fatigue, and cognitive impairment, in this optimized QuilA-EAE model. We made an in-depth characterization of this optimized QuilA-EAE model, describing for the first time somatic thermal hyperalgesia associated with mechanical and cold allodynia. Evaluation of orofacial pain sensitivity showed no mechanical or thermal allodynia. Detailed evaluation of motor behaviors highlighted slight defects in fine motor coordination in the QuilA-EAE mice but without impact on pain evaluation. Finally, no anxiety-related or cognitive impairment was observed during the peak of sensitive symptoms. Pharmacologically, as previously described, we found that pregabalin, a treatment commonly used in neuropathic pain patients, induced an analgesic effect on mechanical allodynia. In addition, we showed an anti-hyperalgesic thermal effect on this model. Our results demonstrate that this QuilA-EAE model is clearly of interest for studying pain symptom development and so could be used to identify and evaluate new therapeutic targets. The presence of interfering symptoms still needs to be further characterized.

## Introduction

Multiple sclerosis (MS) is the most frequent chronic disease that generates neurological disability in young adults. It affects some 2–3 million people worldwide (1). Although the etiology of MS is still unknown, its pathological hallmark is demyelinated lesions in the central nervous system (CNS) white matter and is associated with a number of other pathological features including inflammation, edema, and axonal damage. Approximately 85% of patients begin with a disease phase called relapsing-remitting MS (RR-MS) during which recurrent and reversible neurological deficits occur. After a median illness period of 20–25 years, RR-MS patients enter a second phase of the disease called secondary-progressive MS (SP-MS), characterized by continuous irreversible neurological decline without relapses. The remaining 15% of patients have a primary progressive disease course (PP-MS). Among the many symptoms (motor, sensory, and cognitive) and types of disabilities associated with MS, chronic pain is a common disabling symptom. Estimates of MS pain prevalence vary widely, ranging from 29 to 86% (2–4) depending on the nature of the pain and the assessment protocols used. One specific meta-analysis reported a prevalence of 63% (5). Pain does not seem to be correlated with disease severity and is heterogeneous in nature, encompassing various forms of nociceptive, neuropathic, or mixed pain conditions (6). Neuropathic pain is more persistent and is one of the most common distressing conditions experienced by patients even in the early stages of the disease (3, 7). Management recommendations for neuropathic pain in MS are similar to those for other causes of neuropathic pain. They include tricyclic antidepressants, serotonin and noradrenaline reuptake inhibitors, and gabapentinoid anticonvulsants used as first-line drug therapy (2, 8, 9). However, randomized, double-blind, and placebo-controlled trials to evaluate the efficacy of these agents in the particular population of MS patients are lacking, and clinical reports suggest that pain in MS is inadequately relieved (10).

Though long neglected, research on pain in MS has gained renewed interest over the last decade, prompting the development of several animal models. Experimental autoimmune encephalomyelitis (EAE) is currently the most commonly used animal model for the study of MS, sharing many clinical and neuropathological features with those observed in patients (11, 12). Thus, it has been naturally used to study pain (13). The EAE model can simulate different disease progressions in which clinical symptoms may occur in a monophasic fashion, either acute or chronic, or in a relapsing form (14). Because experiments studying pain in mice very often use nociceptive stimuli and assess induced behavioral responses, severe motor impairments are confounding factors. To address this issue, novel variants of the EAE model have been developed with appropriate modifications to the immunization protocol. Khan et al., described an optimized RR-EAE-mouse model of MS-induced neuropathic pain (QuilA-EAE) using MOG_35−55_ and pertussis toxin for mice immunization in which the conventional complete Freund adjuvant (CFA) was replaced by Quil A (15). This replacement overcame the disadvantages of CFA, which include development of severe motor symptoms (12, 16), reactions at the injection site (17), and neuroinflammatory changes in the CNS (18, 19). Quil A induces proliferation of both B and T cells while preferentially inducing T-cell expansion without persistence at the site of injection. With this protocol, mice exhibited a mild RR clinical disease course with temporal development of mechanical allodynia in both hindpaws, fully developed by 28–30 days post-immunization and maintained until study completion; no confounding motor deficit was observed (15, 20). To our knowledge, the assessment of pain in this model has been limited to the evaluation of mechanical and cold somatic allodynia, whereas in patients with MS the situation is more complex (6). In fact, MS patients display a variety of sensory symptoms, such as spontaneous pain, evoked pain (allodynia and hyperalgesia), dysesthesia, and hypoesthesia. Furthermore, compared to other neurological conditions, pain has greater interference in MS. Pain is strongly associated with fatigue, mood disturbances, such as depression and anxiety, and sometimes cognitive impairment (4, 6, 21–24).

In line with these clinical observations, we carried out an exhaustive analysis of behavioral defects elicited in this QuilA-EAE model. Our purpose was to propose a better characterization and evaluate its construct validity (i.e., the strength of the relationship between experimental systems and the human scenarios they are intended to simulate). We thus (i) characterized sensitive dysfunction, evaluating both somatic and orofacial behaviors, (ii) extended the behavioral characterization to cognitive impairment, fatigue and mood disturbances, and (iii) evaluated the efficacy of a first-line drug for the treatment of neuropathic pain, namely, pregabalin.

## Materials and Methods

### Animals

All experiments were carried out in accordance with the European Communities Council Directive of September 22, 2010 (2010/63/EU) and its application in French law (Decree No. 2013-118 of 1 February 2013) with the approval of the local ethics committee (APAFIS-4306). They conformed to the ethics guidelines of the International Association for the Study of Pain (25) and the Animal Research: Reporting *in vivo* Experiments (ARRIVE) guidelines on reporting standards for pre-clinical studies (26).

Female C57BL/6J mice aged 4 weeks were purchased from Janvier Labs (Le Genest-Saint-Isle, France). A total of 162 females, 81 control mice (CTL), and 81 immunized mice (EAE) were used for this study. Mice were housed six per cage (three CTL, three EAE) in a temperature-controlled environment (22°C ± 2) under a 12:12 light/dark cycle (light from 7:00 a.m. to 7:00 p.m.), with *ad libitum* access to food and water. They were acclimatized for at least 7 days before the start of the experiment. All tests took place during the light phase between 8:00 a.m. and 12:00 a.m. for the open field, rotarod, grip strength, von Frey, air puff, and brush tests, and between 2:00 p.m. and 6:00 p.m. for elevated plus maze, Y-maze, marble burying, hotplate, acetone paw, and acetone face evaporation tests. Throughout the study, EAE scores and body weight were evaluated daily.

### Chemicals and Drugs

The chemicals and drugs used in this study were cx myelin oligodendrocyte glycoprotein peptide human fragment MOG_35−55_ MEVGWYRPPFSRVVHLYRNGK (Sigma Aldrich, Darmstadt, Germany), saponin from quillaja bark (Quil A; Sigma Aldrich, Darmstadt, Germany), pertussis toxin (PTX; Sigma Aldrich, Darmstadt, Germany), pregabalin [(S)-(+)-3-(aminomethyl)-5-methylhexanoic acid (Zhejiang Chiral Medicine Chemicals Co, Ltd, Hangzhou, China], and acetone (Laboratoires Humeau, La Chapelle-sur-Erdre, France). All drugs were dissolved in injectable sterile 0.9% sodium chloride (NaCl, CDM Lavoisier, Paris, France).

### EAE Induction and Assessment

Following the protocol described by Khan et al. (15), mice were immunized using 100 μl of sterile NaCl containing 200 μg of MOG_35−55_ and 45 μg of saponin Quil A. These were administered in four subcutaneous injections (25 μl) in both flanks and shoulder regions. An intraperitoneal (i.p.) injection of 250 ng PTX was administered just after the subcutaneous injection and 48 h later. Control animals were injected with Quil A (45 μg) and PTX (250 ng) alone. Mice were injected by an independent experimenter (MB) different from those performing the rest of the study (AD) to prevent experimental bias.

Mice were monitored daily for clinical score and body weight. For clinical scoring, we used the five-point scale with half gradations established by Khan et al. (15) (0: normal behavior, 0.5: limpness of the distal tail region and hunched appearance, 1: completely limp tail or developing weakness in the hindlimbs, 1.5: lip tail and distinct hindlimb weakness, 2: limp tail with unilateral partial hindlimb paralysis, 2.5: limp tail and partial paralysis of bilateral hindlimbs, 3: complete paralysis of bilateral hindlimbs, 3.5: complete bilateral hindlimb paralysis and unilateral forelimb paralysis, and 4: quadriplegia). The previously established half-point scale enabled us to monitor and record gradual behavioral and physical changes that occurred over the course of the study (27). The EAE disease was regarded as present if clinical scores were ≥1. Clinical scores ≤ 0.5 were indicative of no disease or disease remission. Clinical scores were evaluated in a blinded manner.

### Behavioral Evaluation

For behavioral studies, QuilA-EAE mice and CTL were tested at disease onset (D17) and during the chronic phase of the disease (D30). In the 14 behavioral procedures, the number of tests for each animal was limited to 4–7 according to the severity of the procedures. Also, to take into consideration the variability of EAE induction (28–30), each behavioral evaluation was always performed in two different cohorts of EAE. Hence, seven cohorts (groups of animals immunized at the same time and evaluated with the same tests) were used (each cohort included 12 CTL and 12 EAE mice except for Cohort 4, in which only 10 animals were included in each group). This procedure is summarized in Table 1. Body weight, RR-EAE score, and von Frey evaluation were determined in all seven cohorts, but for simplicity, we show data from only two cohorts closely representative of the others. All behavioral assessments were conducted by an experimenter blind to the immunization status.

**Table 1:** Summary table of tests performed by cohorts.

**Test**	**Cohort 1** **(*n* = 12/group)**	**Cohort 2** **(*n* = 12/group)**	**Cohort 3** **(*n* = 12/group)**	**Cohort 4** **(*n* = 10/group)**	**Cohort 5** **(*n* = 12/group)**	**Cohort 6** **(*n* = 11/group)**	**Cohort 7** **(*n* = 12/group)**
General observations	Body weight EAE scoring	Body weight EAE scoring					
Motor			Rotarod Open field Grip test	Rotarod Open field Grip test	Grid test	Grid test	-
Sensitive	–	Acetone facial	Von Frey pharmaco Hotplate Acetone paw test	Von Frey Chaplan Hotplate Acetone paw test	Von Frey Chaplan Paintbrush test Air puff	Acetone facial Paintbrush test Air puff	Hotplate pharmaco
Cognitive	Object recognition Social interaction	Object recognition EPM Marble burying Y-maze	Y-maze EPM Marble burying	–	–	Social interaction	-

*Body weight, Experimental Autoimmune Encephalomyelitis (EAE) scoring, and von Frey Chaplan were determined in all seven cohorts. Here we show data from only two cohorts closely representative of the others*.

#### Motor Evaluation

**Spontaneous locomotion** activity was evaluated using the open field test. Mice were placed in the center of a white polyvinyl chloride open field apparatus (50 cm long × 50 cm wide × 45 cm high) and allowed to move freely. Virtual areas, a central square (30 cm long), and a peripheral zone were determined with videotracking software (Viewpoint, Lyon, France). For 15 min, total distance traveled and time spent in the central area were recorded with the videotracking system.

**Motor coordination** was determined using a standard mouse rotarod (TSE, Bad Homburg Germany). We used the standard operating procedures described by the EUMORPHIA group program (31). Briefly, after a training phase to identify mice able to stay on the rod at 4 rpm for 60 s, mice underwent the test phase. This consisted of four trials with a 15 min intertrial interval. In each trial (T1–T4), mice were placed on the rod rotating at 4 rpm, the timer was started, and the rod was accelerated from 4 to 40 rpm in 300 s. The latency to fall off the rod was determined automatically. However, the timer was manually stopped if a mouse held onto the rod in a full rotation (“passive rotation”).

**Ataxic behaviors** or **fine motor coordination** were evaluated using the grid test adapted from Belknap (32). The grid test apparatus consisted of two 50 cm long × 50 cm wide × 50 cm high white acrylic boxes placed in the center of a videotracking system (Phenorack, Viewpoint, Lyon, France). Two cameras were used, one mounted on the roof (top view of mice) and another on the wall (side view of mice). One side of the white acrylic boxes was made of transparent acrylic to allow the side recording of mice by the second camera. Mice were placed on a 50 cm long × 50 cm wide wire grid floor (wire diameter 1.5 mm, grid mesh 1 × 1 cm) 30 cm above an infrared floor. Locomotor activity was recorded by the first camera. The experiment was stopped when the mice had traveled a distance of 300 cm. The number of times that mice slipped on the wire mesh floor was counted by an experimenter from recordings made by the second camera.

**Muscular strength** was measured for forelimbs or both hind and forelimbs with a grip strength meter (Bioseb, Chaville, France). We used the standard operating procedure described by the EUMORPHIA group program (31). Mice were lifted and held by their tails so that their limbs could grasp a wire grid. The mice were then gently pulled backward by their tails with their posture parallel to the surface of the bench until they released the grid. The grip strength meter digitally displayed the maximum force applied as the peak tension (in newtons) when grasp was released. Each mouse performed five consecutive trials, first with forelimbs and then with both hind and forelimbs. The mean of the five trials was taken as an index of limb grip strength.

#### Somatic Evaluation of Pain Sensitivity

**Mechanical sensitivity** was evaluated using the von Frey test by the method described by Chaplan et al. (33) and modified by Dixon (34). Mice were placed in individual plastic boxes (3.5 cm long × 8 cm wide × 14 cm high) on an elevated mesh platform, allowing full access to the paws. Mice were habituated to the apparatus for 45–60 min before paw withdrawal threshold measurement (PWT). Stimulation was applied using the up-and-down method. Calibrated von Frey filaments in the range 0.02–1.4 g (Bioseb Aesthesio®, Chaville, France) were applied perpendicularly to the right hindpaw with sufficient force to cause a slight buckling against the paw for 3–5 s. A positive response corresponds to a paw withdrawal, flinching, or licking. The PWT was determined as previously described (33).

**Heat hyperalgesia** was assessed using the hotplate test (35). The reaction threshold to a high-intensity heat stimulus was measured as an index of peripheral pain response. Mice were placed on a square metal surface heated to a temperature of 52 or 56°C (model-DS 37, Ugo Basile, Gemonio, Italy), and latencies to the first nocifensive response is characterized by the following signs: licking, shaking hindpaws, or jumping. As soon as the nocifensive response was observed, timer was stopped and the mouse was immediately removed from the hot plate. Data validation requisites the getting of two stable latencies (<1 s of difference) with a maximum of four trials performed per mouse. Data that did not fulfill this validation criterion were exclude. Cut-off latencies of 30 s (for 52°C) and 15 s (for 56°C) were used to prevent paw injury.

**Cold allodynia** was evaluated using the acetone evaporation test adapted from Chen et al. (36). Mice were placed in individual plastic boxes (3.5 cm long × 8 cm wide × 14 cm high) on an elevated mesh platform, allowing full access to the paws. Before stimulation, mice were habituated to the box for 30–45 min. A drop (20 μl) of acetone (Laboratoires Humeau, La Chapelle-sur-Erdre, France) was laid on the plantar surface of the hindpaw without touching the skin with the dropper tip, and the response was observed for 60 s after acetone application. Responses to acetone were scored as 0: no response, 1: quick withdrawal, flick, or stamp of the paw, 2: prolonged withdrawal or repeated stamping or flicking of the paw, 3: licking of the paw, or 4: jumping. The nocifensive score was the sum of all the responses evoked in the 60 s after acetone application. Acetone was alternately applied three times to each hindpaw. Because we did not manipulate one particular side of the CNS, an average of the six nocifensive scores was calculated for each mouse.

#### Orofacial Evaluation of Pain Sensitivity

Given that some patients with MS experience trigeminal neuralgia [3.8%, according to Foley et al. (5)], we investigated orofacial sensitivity in the QuilA-EAE model.

**Dynamic mechanical sensitivity** was assessed using the paintbrush test adapted from Thibault et al. (37). Mice were allowed to move freely in individual boxes (3.5 cm long × 7.5 cm wide × 8 cm high) made of plexiglass and wire mesh for the front side allowing full access to the face and thus reducing any restraint effect. Prior to stimulation, the mice were habituated to the box for 30–45 min. Two paintbrushes were used to rub whisker pads, namely, a very smooth one made of marten hairs and a rough one made of pig bristles. The stimulus was applied five times to each whisker pad. The number of positive responses (quick head withdrawal, escape, or attempt to attack the paintbrush) was noted for each side. Because we did not manipulate one particular side of the CNS, scores for the left and right whisker pad were averaged for each mouse.

**Static mechanical sensitivity** was determined using the air puff test using a protocol adapted from Thorburn et al. (38). Mice were allowed to move freely in individual boxes (3.5 cm long × 7.5 cm wide × 8 cm high) made of plexiglass and wire mesh for the front side, allowing full access to the face and reducing any effects of restraint. Prior to stimulation, the mice were habituated to the box for 30–45 min. Three different air puff stimuli were applied to the whisker pad 1 cm away from the skin to avoid physical contact. Each air puff stimulus applied to the face was characterized according to its duration and maximal intensity perceived at the stimulation site [Table 2, for comparison stimulation of 10 psi described in Thorburn et al. (38) elicited 25.82 mpsi in our quantification system of maximal intensity perceived]. For each air puff stimulus, three stimulations were applied to the right and the left mouse whisker pad and nociceptive responses were scored. Between each air puff, there was a resting time of 30 min. Scoring was as follows: 0, no response; 0.25: brisk withdrawal of the head from the stimulus probe or an attempt to attack the probe; 1, single unilateral or bilateral forepaw swipe down the snout; and 1.5, continuous unilateral or bilateral forepaw swipe down the snout (three or more). Because we did not manipulate one particular side of the CNS, a total nociceptive response was calculated by averaging the scores for the left and right whisker pad for each mouse. The reproducibility of air puffs for each type of stimulation was determined using a force transducer MLTF500/ST device (ADInstruments Ltd, Paris, France) after 60 consecutive stimulations (Supplementary Figure S1).

**Table 2:** Summary table of air puff stimulation characteristics.

**Stimulation**	**1**	**2**	**3**
Duration (s)	0.16	0.14	0.48
Max intensity (mpsi)	1.8	23.0	26.4

**Sensitivity to cold** was evaluated using the facial acetone evaporation test adapted from Constandil et al. (39). Mice were acclimated for 15 min in a glass chamber with three mirrored sides (back and sides) and one transparent side (30 cm long × 30 cm wide × 30 cm high). Mice were gently restrained to allow full access to the face. A drop (20 μl) of acetone was laid on the left side of the face without touching the skin with the dropper and the mice were immediately returned to the glass chamber. The duration of facial rubbing or scratching behavior evoked by vaporization of acetone was counted for 1 min after acetone application.

#### Cognitive Evaluation

To achieve this behavioral characterization, we evaluated cognitive deficit given that 20–25% of patients with RR-MS at disease onset suffer from cognitive impairment, which most frequently affects processing speed and memory (22, 23).

**Sociability and social novelty preference** were evaluated using an adapted protocol of the three-chamber sociability and social novelty test (40). The test comprised three sessions. Between each session, the mouse was returned to its home cage for 5 min. In the habituation session, the mouse was placed in the middle of a white polyvinyl chloride open field (50 cm long × 50 cm wide × 45 cm high) containing two empty wire cups (an inverted stainless steel wire pencil cup; Galaxy, Kitchen Plus) on both sides. A heavy cup was placed on the top of each inverted wire pencil cup to prevent the subject from climbing on top or moving it. In a first session, mice were allowed to explore freely for 5 min. Exploration times and movements of the mice were recorded using a videotracking system (Viewpoint, Lyon, France). Virtual zones were defined to delimitate the three chambers and the area of exploration of the wire cups. In the T1 session (5 min duration), an unfamiliar adult WT female was placed in one of the wire cups. This session allowed the evaluation of the sociability index (exploration time of unfamiliar mice—exploration time of empty cup / total exploration time of both cups). In the T2 session (duration 5 min), while the first intruder was still posted in its wire cup, another unfamiliar adult WT female was placed in the second cup. A novelty percentage was then calculated [(exploration time of unfamiliar mice/total exploration time of both mice) × 100].

**Episodic memory** was assessed using the novel object recognition test (41). The test comprised two sessions. During the first session T1, the mouse was placed in a white polyvinyl chloride cage (36 cm long × 14 cm wide × 21 cm high) on a layer of sawdust and was presented with two identical objects placed at the two opposite sides of the cage. The session was stopped after 20 s of object exploration. A cut-off time was fixed at 5 min and mice that had not performed this exploration were excluded from the study. Between T1 and T2, the mouse was moved back to its home cage for 10 min. In the second session T2, one object was replaced by a new object, and for 5 min, the exploration time for each object was measured. A recognition percentage was then calculated [(exploration time of new object/total exploration time of both objects) × 100].

**Working memory** was evaluated using the Y-maze test according to the standard operating procedure described by the EUMORPHIA group program (31). Spontaneous alternation behavior and exploratory activity were recorded. The apparatus was made of three equal arms (40 cm long × 10 cm wide × 16 cm high) made of black polyvinyl chloride radiating at 120° from each other. Mice were placed at the end of one arm, facing the end wall of the arm, and allowed to move freely through the maze for a 10 min session. Latency to leave the first arm and total number and sequence of entries into each arm were scored for each mouse. An arm entry was counted when the mouse had all four paws inside the arm. If mice completed fewer than eight arm entries within 10 min, they were excluded from further analysis. Spontaneous alternation was defined as entries into all three arms on three consecutive choices. The alternation score (%) represents an index of working memory and was calculated as follows: % alternation = 100 × [number of alternations/(total arm entries – 2)].

**Anxiety** was assessed using the elevated plus maze (EPM), which assesses the natural conflict between the tendency of mice to explore a novel environment and their tendency to avoid a brightly lit elevated open area (42). The EPM comprised of two open arms and two closed arms (37 cm long × 6 cm wide) that extended from a central platform (6 cm × 6 cm). The apparatus was constructed of Plexiglas (black floor and walls) and elevated at 50 cm above floor level. The mouse was placed on the central platform facing one of the enclosed arms and was allowed to explore the maze for 10 min. The number of open arm entries, the time spent in different parts of the maze (open and closed arms, central platform), and the total number of open and closed arm entries were recorded with a videotracking system (Viewpoint, Lyon, France). Mice completing fewer than eight arm entries within 10 min were excluded from further analysis.

**Anxiety** was also evaluated using the marble burying test adapted with minor modifications from Millan et al. (43). Mice were individually placed in polyvinyl chloride cages (36 cm long × 21 cm wide × 14 cm high) containing a 4 cm layer of sawdust and 20 glass marbles (diameter 1.6 cm) that were evenly spaced throughout the cage (five rows of four marbles). Mice explored the cage freely for 30 min. The number of marbles buried more than two thirds into the sawdust was counted at 30 min.

### Pharmacological Impact of Somatic Sensitivity

A previous study by Khan et al. (15) in the QuilA-EAE model showed an analgesic effect of gabapentin and amitriptyline on mechanical hypersensitivity. We evaluated the analgesic potential effect of pregabalin, another recommended treatment for neuropathic pain management (9).

The analgesic effect of pregabalin was tested when mechanical hypersensitivity and thermal hyperalgesia were fully developed from D30 to D40 during the chronic phase of the disease. Mice received an i.p. injection with a vehicle (sterile NaCl for injection) of a single bolus of pregabalin at 10 or 30 mg/kg. Drugs were prepared just before the injections (MB) and administered according to the block method to assess their effect in the same conditions. Each mouse received up to one vehicle injection and two doses of a single drug with which each mouse received in a different order. There was a washout period of 4 days between successive doses. Throughout the protocol, the experimenter (AD) was blind to mice immunization status and treatments.

**The analgesic effect of pregabalin on mechanical hypersensitivity** was evaluated using the von Frey frequency method. As described previously, mice were placed in individual plastic boxes on an elevated mesh platform and allowed to habituate for 45–60 min. The withdrawal frequency was determined using von Frey's hair filaments with two different bending forces (0.4 and 1.4 g) before injection (baseline) and 15, 30, 60, 90, and 120 min post-injection. The filaments were applied perpendicularly to the plantar surface of the hindpaw until they buckled. For each filament, five stimuli were applied with an interval of 3–5 s to the right and left hindpaws. Withdrawal frequency (%) was quantified as number of withdrawals observed for the right and left hindpaws/total number of stimulations × 100.

**The analgesic effects of pregabalin on heat hyperalgesia** was assessed using the hotplate test at 56°C as described previously. For baseline, an average of two nocifensive latencies of <1 s was calculated. For *t* = 15, 30, 60, 90, and 120 min, one value of latency was measured per time point. To avoid paw injury, a cut-off was set at 15 s.

### Statistical Analysis

All statistical analyses were carried out using GraphPad Prism 6 software (GraphPad Inc., San Diego, USA).

Before each analysis, equality of variance and normal distribution were evaluated, and parametric or non-parametric analyses were performed according to the results observed.

For body weight and EAE score longitudinal monitoring, data were analyzed using two-way ANOVA with group (CTL vs. QuilA-EAE) and day post-induction (DPI) (D0–D40) as main factors with DPI being defined as a repetitive measure. *Post-hoc* comparisons were made with a Sidak test for multiple comparisons between groups for each DPI. For motor and sensitivity behavioral tests, data were analyzed using two-way ANOVA with group (CTL vs. QuilA-EAE) and DPI (D17 vs. D30) as main factors, with DPI being defined as a repetitive measure. *Post-hoc* comparisons were made with a Sidak test for multiple comparisons between groups for each time point.

For the air puff test, data were analyzed using two-way ANOVA with group (CTL D17, CTL D30, EAE D17, EAE D30) and air puffs (stimulation 1, 2, 3) as main factors. *Post-hoc* comparisons were made with a Sidak test for multiple comparisons between CTL and EAE mice for each air puff stimulation.

For cognitive behavioral tests at D30, statistical analysis was carried out using unpaired parametric (two-tailed) Student *t-*test (CTL vs. QuilA-EAE). In cases where assumptions of normality criteria (EPM Cohorts 2 and 3) or equality of variance (object recognition Cohort 1) were not met, the Mann-Whitney test was performed (CTL *vs*. QuilA-EAE).

For pharmacological studies, for each group (CTL vs. QuilA-EAE), areas under the curves (AUC) of the kinetics of treatment effect (0–120 min) were calculated using the trapezoidal rule and analyzed using a one-way ANOVA with treatment as main factors. *Post-hoc* comparisons were made with Tukey's test.

Results were presented as mean ± standard error of the mean (SEM). A *p*-value < 0.05 was taken as the statistical significance level.

## Results

### Behavioral Evaluation

#### General Aspect and Disease Time Course

**Figure 1:**
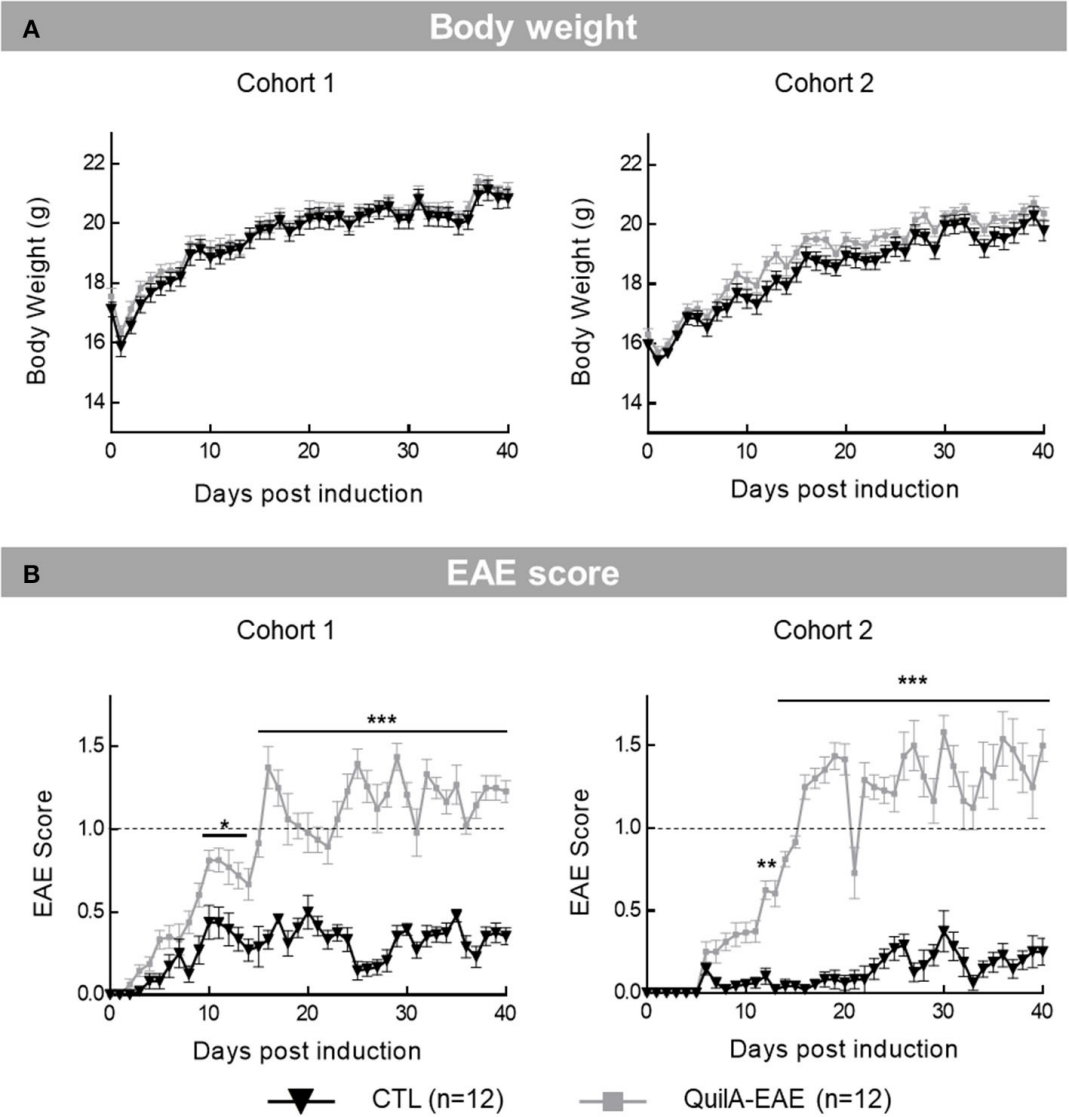
Longitudinal monitoring of body weight and clinical scores in mice immunized with MOG_35−55_ (QuilA-EAE) and their controls (CTL) from post-induction Day 0 (D0) to D40. **(A)** Time course of body weight. Results are means ± SEM. **(B)** Time course of EAE clinical score based on physical observation defined by Khan et al. (15) ranging from 0 (normal behavior) to 4 (quadriplegia). Results are means ± SEM. The dashed line represents level of motor defects considered as clinically relevant score (EAE score 1: completely limp tail or hindlimb weakness). Statistical analysis was performed using two-way ANOVA followed by *post-hoc* Sidak test; ^**^p < 0.01, ^***^p < 0.001 vs. control (shown for Sidak test only). ^*^p < 0.05.

In Cohorts 1 and 2, the two-way ANOVA of **body weight** showed a significant main effect of DPI [*F*_(40,880)_ = 265.4, *p* < 0.0001 and *F*_(40,880)_ = 221.5, *p* < 0.0001 in Cohorts 1 and 2, respectively] but not group [*F*_(1,22)_ =0.4261, *p* > 0.05 and *F*_(1,22)_ = 2.718, *p* > 0.05 in Cohorts 1 and 2, respectively] or DPI × group interaction [*F*_(40,880)_ = 0.9247, *p* > 0.05 and *F*_(40,880)_ = 0.910, *p* > 0.05 in Cohorts 1 and 2, respectively]. For the entire duration of the study, no body weight difference was shown by *post-hoc* analysis comparing groups (Sidak test, *p* > 0.05). *Post-hoc* comparing DPI showed an increase in body weight with time (Sidak test, *p* < 0.05) (Figure 1A).

For **EAE clinical score**, two-way ANOVA in Cohorts 1 and 2 showed a significant main effect of DPI [*F*_(40,880)_ = 27.15, *p* < 0.0001 and *F*_(40,880)_ = 30.00, *p* < 0.0001 in Cohorts 1 and 2, respectively], group [*F*_(1,22)_ = 201.3, *p* < 0.001 and group *F*_(1,22)_ = 291.9, *p* < 0.0001 in Cohorts 1 and 2, respectively] and DPI × group interaction [*F*_(40,880)_ = 11.53, *p* <0.0001 and *F*_(40,880)_ = 18.34, *p* < 0.001 in Cohorts 1 and 2, respectively]. *Post-hoc* analysis showed a significant increase in EAE score between mice immunized with MOG_35−55_ (EAE) and their controls (CTL) from D10 to D40 in Cohort 1 (Sidak test, D10–D14 *p* < 0.05; D15–D40 *p* < 0.001) and from D12 to D40 in Cohort 2 (Sidak test, D12–D40 *p* < 0.001). In both Cohorts 1 and 2, modeled disease onset started at D16 when EAE mice reached a score ≥1. Behavioral evaluation was assessed at D17 (beginning of disease) and D30 (disease fully installed). For each cohort, a mean EAE clinical score higher than 2 (limp tail with unilateral partial hindlimb paralysis) was never observed. Overall, the time course of the EAE score reproduced the clinical presentation of the QuilA-EAE model with alternating phases of relapse and remission (Figure 1B).

#### Motor Evaluation

##### QuilA-EAE Mice Exhibited No Locomotion, Motor Coordination, or Muscular Strength Defects

**Figure 2:**
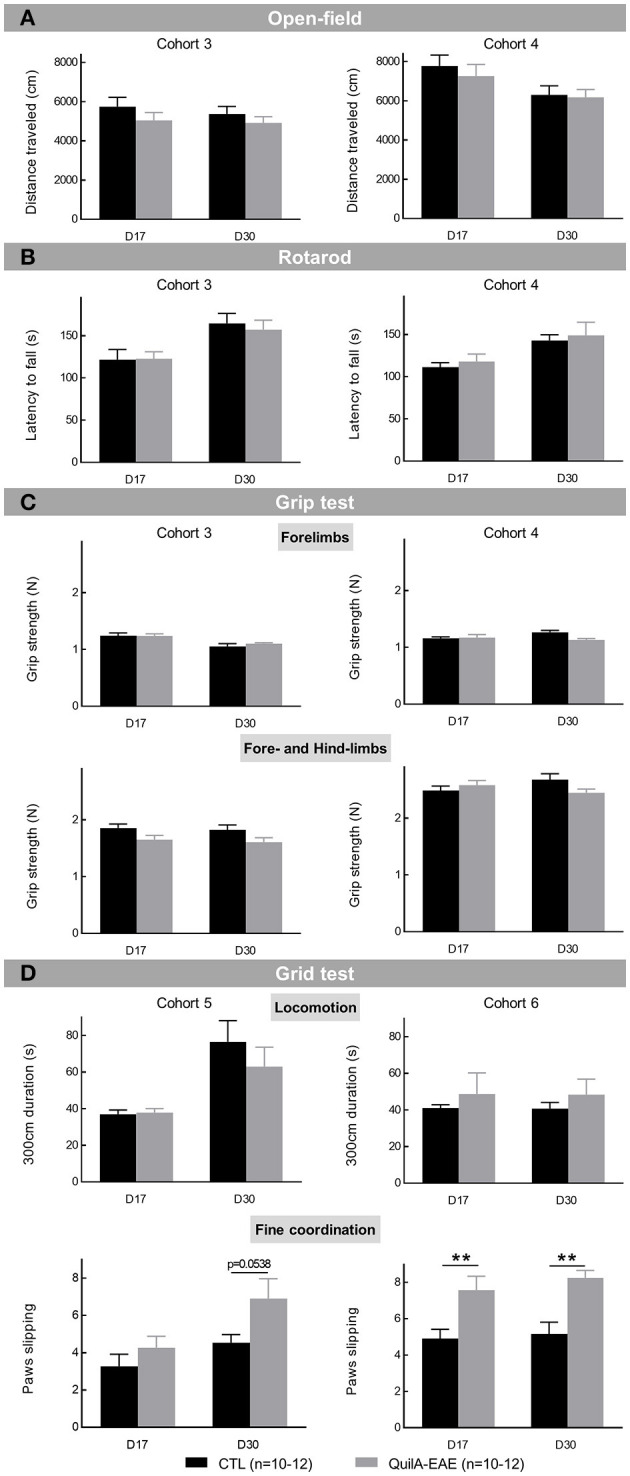
Evaluation of motor behaviors in mice immunized with MOG_35−55_ (QuilA-EAE) and their controls (CTL) at post-induction Day 17 (D17) and D30. **(A)** Evaluation of spontaneous locomotor activity using the open-field test. Locomotion was evaluated using the total distance traveled ± SEM for the overall 15 min session. **(B)** Rotarod performance was expressed as the mean latency to fall ± SEM for the four rotarod sessions performed by each mouse. **(C)** Muscular strength performance was expressed as the mean grip strength ± SEM for the five sessions performed by each mouse. Top: forelimb muscular strength. Bottom: fore and hindlimb muscular strength. **(D)** Evaluation of fine coordination using the grid test. Top: locomotion was evaluated using time to travel the 300 cm required distance ± SEM. Bottom: fine motor coordination was assessed using the number of paw slips on holes in the grid ± SEM for a distance of 300 cm. Statistical analysis was performed using two-way ANOVA followed by *post-hoc* Sidak test; ^**^p < 0.01 vs. control (shown for Sidak test only).

**Motor behavior** was evaluated by assessing spontaneous locomotor activity. The two-way ANOVA of the total distance traveled in the open field showed a main significant effect of DPI in Cohort 4 [*F*_(1,18)_ = 9.951, *p* < 0.01] but was not observed in Cohort 3 [*F*_(1,22)_ = 1.284, *p* > 0.05]. In Cohorts 3 and 4, no effect of group [*F*_(2,22)_ = 1.238, *p* > 0.05 and *F*_(2,18)_ = 0.2826, *p* > 0.05 in Cohorts 3 and 4, respectively] and DPI × group interaction [*F*_(1,22)_ = 0.3027, *p* > 0.05 and *F*_(1,18)_ = 0.2452, *p* > 0.05] was observed. *Post-hoc* analysis showed no difference in total locomotor activity of QuilA-EAE compared to CTL mice at D17 and D30 in Cohort 4 (Figure 2A).

**Motor coordination** was assessed by evaluating latency to fall from the rotarod. The two-way ANOVA showed a significant main effect of DPI [*F*_(1,22)_ = 74.57, *p* < 0.0001 and *F*_(1,18)_ = 23.89, *p* < 0.0001 in Cohorts 3 and 4, respectively] but no effect of group [*F*_(1,22)_ = 0.0443, *p* > 0.05 and *F*_(1,18)_ = 0.2590 *p* > 0.05 in Cohorts 3 and 4, respectively] and of DPI × group interaction [*F*_(1,22)_ = 0.9075 *p* > 0.05 and *F*_(1,18)_ = 0.0002, *p* > 0.05 in Cohorts 3 and 4, respectively]. *Post-hoc* analysis showed no difference in motor coordination between QuilA-EAE and CTL mice at D17 and D30 (Figure 2B).

**Muscular strength** was measured for forelimbs or both fore and hindlimbs. For **forelimbs**, the two-way ANOVA showed a significant main effect of DPI in Cohort 3 [*F*_(1,22)_ = 31.50, *p* < 0.0001] but was not observed in Cohort 4 [*F*_(1,18)_ = 0.7618, *p* > 0.05]. No group effect [*F*_(1,22)_ = 0.2006, *p* > 0.05 and *F*_(1,18)_ = 3.007, *p* > 0.05 in Cohorts 3 and 4, respectively] and no DPI × group interaction [*F*_(1,22)_ = 0.7961, *p* > 0.05 and *F*_(1,18)_ = 3.884, *p* > 0.05 in Cohorts 3 and 4, respectively] was observed (Figure 2C, **top**). For **fore and hindlimbs**, the two-way ANOVA showed a significant main effect of group in Cohort 3 [*F*_(1,22)_ = 5.413, *p* < 0.01] but not in Cohort 4 [*F*_(1,18)_ = 0.5087, *p* > 0.05]. No DPI effect [*F*_(1,22)_ = 0.3985, *p* > 0.05 and *F*_(1,18)_ = 0.1750, *p* > 0.05 in Cohorts 3 and 4, respectively] was observed. A significant DPI × group interaction was observed in Cohort 4 [*F*_(1,18)_ = 0.6.004, *p* < 0.05] but not in Cohort 3 [*F*_(1,22)_ = 0.0111, *p* > 0.05]. *Post-hoc* analysis showed no difference in muscular strength between QuilA-EAE and CTL mice at D17 and D30 (Figure 2C, **bottom**).

Optimized relapsing-remitting experimental autoimmune encephalomyelitis (QuilA-EAE) mice thus exhibited neither a decrease in spontaneous locomotor activity nor a defect in motor coordination and muscular strength, demonstrating the absence of a motor confounding effect during sensory evaluation.

##### QuilA-EAE Mice Exhibited Fine Motor Coordination Impairment

**Fine motor coordination** was determined using the grid test in Cohorts 5 and 6. When the time taken to travel a distance of 300 cm was analyzed, the two-way ANOVA showed no effect of group [*F*_(1,20)_ = 0.5946, *p* > 0.05 and *F*_(1,22)_ = 0.5766, *p* > 0.05 in Cohorts 5 and 6, respectively] or any DPI × group interaction [*F*_(1,20)_ = 0.8593, *p* > 0.05 and *F*_(1,22)_ = 0.0006, *p* > 0.05 in Cohorts 5 and 6, respectively]. In Cohort 5, a significant main effect of DPI [*F*_(1,20)_ = 17.34, *p* < 0.001] was shown. It was also not observed in Cohort 6 [*F*_(1,22)_ = 0.0209, *p* > 0.05]. *Post-hoc* analysis showed no difference in the time taken to travel a distance of 300 cm between QuilA-EAE and CTL mice at D17 and D30, showing the absence of strong locomotor dysfunction in QuilA-EAE mice (Figure 2D, **top**).

When the number of paw slips was analyzed, the two-way ANOVA showed a significant main effect of group [*F*_(1,20)_ = 5.290, *p* < 0.05 and *F*_(1,22)_ = 23.90, *p* < 0.0001 in Cohorts 5 and 6, respectively]. In Cohort 5, a significant main effect of DPI [*F*_(1,20)_ = 7.223, *p* < 0.05] was shown, but was not observed in Cohort 6 [*F*_(1,22)_ = 0.5749, *p* > 0.05]. No DPI × group interaction was observed [*F*_(1,20)_ = 0.8789, *p* > 0.05 and *F*_(1,22)_ = 0.1188, *p* > 0.05 in Cohorts 5 and 6, respectively]. In Cohort 6, *post-hoc* analysis showed a significant increase in paw slips in QuilA-EAE mice compared to CTL at D17 (disease onset) and D30 (during the chronic phase of the disease) (Sidak test, *p* < 0.01 for both). In Cohort 5, *post-hoc* analysis showed no statistical difference, although a strong trend of increase in paw slips in QuilA-EAE mice compared to CTL was observed at D30 (Sidak test, *p* = 0.0538) (Figure 2D, **bottom**). These results strongly suggest that QuilA-EAE exhibited weak impairment of fine motor coordination at least at D30.

#### Evaluation of Somatic Pain Sensitivity

##### QuilA-EAE Mice Developed a Mechanical Allodynia

**Figure 3:**
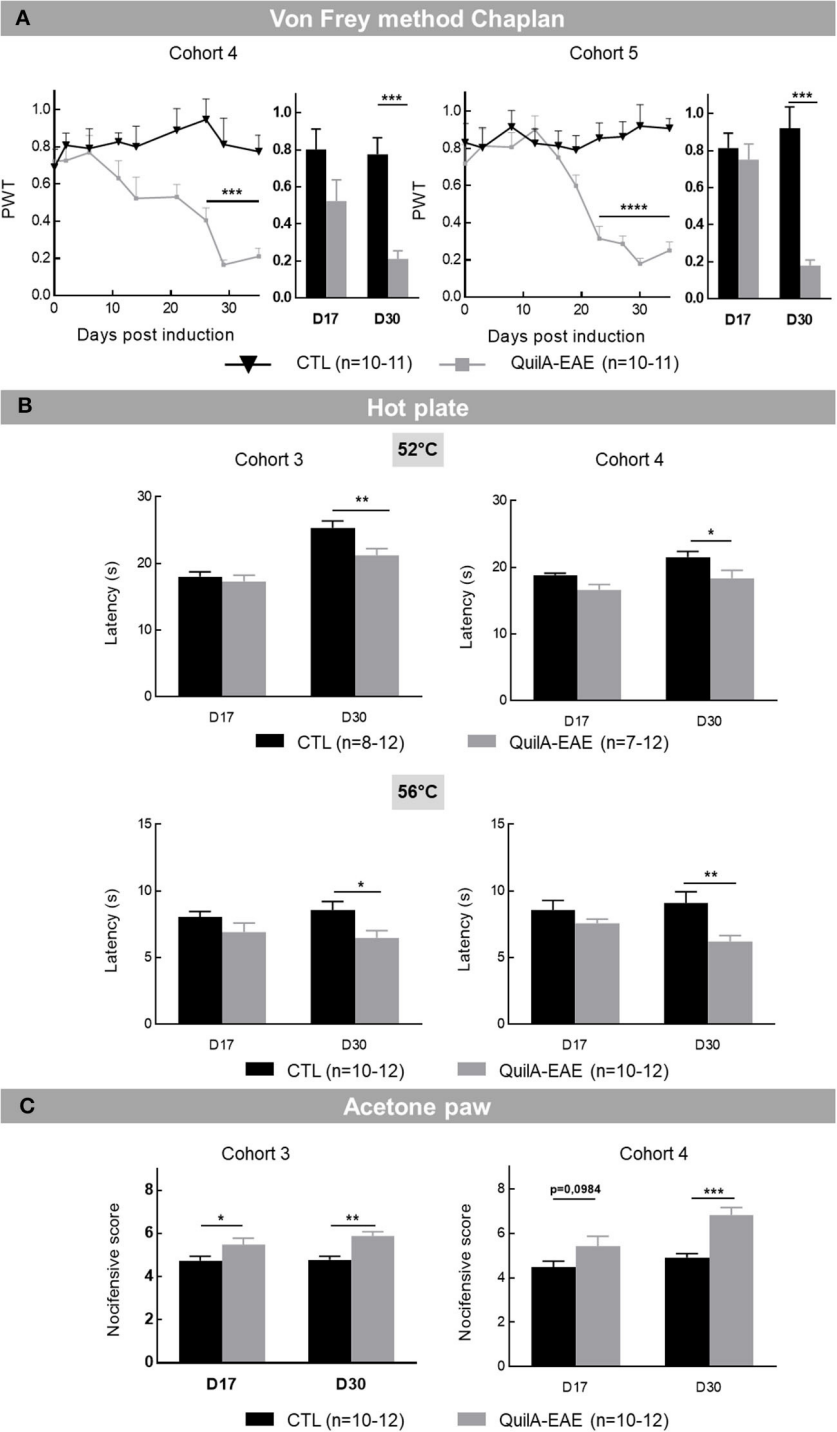
Evaluation of somatic sensitivity in mice immunized with MOG_35−55_ (QuilA-EAE) and their controls (CTL) at post-induction Day 17 (D17) and D30. **(A)** Mechanical sensitivity from post-induction Day 0 (D0) to D35. Mechanical sensitivity expressed as the mean of paw withdrawal threshold (PWT) ± SEM for each time point. Data are illustrated using time course curves and bar chart showing results obtained at D17 and D30. **(B)** Heat hyperalgesia at D17 and D30 expressed as the mean time taken to observe a nocifensive behavior in mice exposed to a hotplate ± SEM with hotplate at 52°C (top) and 56°C (bottom). **(C)** Cold allodynia at D17 and D30 was expressed as the mean number of nociceptive responses ± SEM observed in mice after acetone drop deposition. Statistical analysis was performed using two-way ANOVA followed by *post-hoc* Sidak test; ^*^p < 0.05, ^**^p < 0.01, ^***^p < 0.001 vs. control (shown for Sidak test only).

Once potentially confounding motor impairment was excluded in the QuilA-EAE model, somatic sensitivity was investigated. First, **mechanical sensitivity** was evaluated in Cohorts 4 and 5 every 3 to 5 days from D0 to D35. The two-way ANOVA of PWT showed a significant main effect of DPI [*F*_(8,144)_ = 3.424, *p* < 0.001 and *F*_(9,180)_ = 5.741, *p* < 0.0001 in Cohorts 4 and 5, respectively], group [*F*_(1,18)_ = 18.48, *p* < 0.001 and *F*_(1,20)_ = 27.71, *p* < 0.0001 in Cohorts 4 and 5, respectively], and DPI × group interaction [*F*_(8,144)_ = 4.644, *p* < 0.001 and *F*_(9,180)_ = 8.358, *p* < 0.0001 in Cohorts 4 and 5, respectively]. *Post-hoc* analysis showed a significant decrease in PWT in EAE mice compared to CTL from D26 to D35 in Cohort 4 (Sidak test, *p* < 0.001) and from D23 to D35 in Cohort 5 (Sidak test, *p* < 0.001) (Figure 3A **time course curves**). Evaluation of PWT at D17 (onset of disease) and D30 (chronic phase of the disease) showed a significant main effect of group [*F*_(1,18)_ = 16.82, *p* < 0.001 and *F*_(1,20)_ = 24.13, *p* < 0.0001 in Cohorts 4 and 5, respectively]. In Cohort 5, main significant effects of DPI [*F*_(1,20)_ = 7.318, *p* < 0.05] and DPI × group interaction [*F*_(1,20)_ = 15.64, *p* < 0.001] were identified. In Cohort 4, no effects of DPI [*F*_(1,18)_ = 4.136, *p* = 0.0570] or DPI × group [*F*_(1,18)_ = 2.2965, *p* > 0.05] were observed. *Post-hoc* analysis showed a strong significant decrease in PWT in EAE mice compared to CTL mice at D30 in each cohort (Sidak test, *p* < 0.001) (Figure 3A **bar chart**). These results show that EAE mice developed a severe and lasting mechanical allodynia fully developed at D30 after disease induction.

##### EAE Mice Developed a Heat Hyperalgesia

After mechanical sensitivity analysis, thermal somatic sensitivity was assessed with heat hyperalgesia evaluation at 52 and 56°C in Cohorts 3 and 4. The two-way ANOVA of the latency of first nocifensive sign at 52°C showed a main effect of DPI [*F*_(1,22)_ = 46.51, *p* < 0.0001 and *F*_(1,13)_ = 10.10, *p* < 0.01 in Cohorts 3 and 4, respectively] and group [*F*_(1,22)_ = 5.620, *p* < 0.05 and *F*_(1,13)_ = 6.754, *p* < 0.05 in Cohorts 3 and 4, respectively] but no DPI × group interaction [*F*_(1,22)_ = 4.164, *p* = 0.0535 and *F*_(1,13)_ = 0.6493, *p* > 0.05 in Cohorts 3 and 4, respectively]. *Post-hoc* analysis showed a significant shortening of latency in EAE mice compared to CTL in each cohort at D30 (Sidak test, *p* < 0.01 and *p* < 0.05 in Cohorts 3 and 4, respectively) but not at D17 (Sidak test, *p* > 0.05) (Figure 3B, **top**). Similarly, at 56°C, the two-way ANOVA showed a main effect of group [*F*_(1,22)_ = 5.701, *p* < 0.05 and *F*_(1,18)_ = 12.28, *p* < 0.01 in Cohorts 3 and 4, respectively] but not DPI [*F*_(1,22)_ = 0.0061, *p* > 0.05 and *F*_(1,18)_ = 0.3467, *p* > 0.05 in Cohorts 3 and 4, respectively] and no DPI × group interaction [*F*_(1,22)_ = 0.3094, *p* > 0.05 and *F*_(1,18)_ = 1.879 sz, *p* > 0.05 in Cohorts 3 and 4, respectively]. *Post-hoc* analysis showed a significant shortening of latency in EAE mice compared to CTL in each cohort at D30 (Sidak test, *p* < 0.05 and *p* < 0.01 in Cohorts 3 and 4, respectively) (Figure 3B, **bottom**). These results show the occurrence of a heat hyperalgesia in QuilA-EAE mice at D30.

##### QuilA-EAE Mice Developed a Cold Allodynia

To complete this somatic sensitivity evaluation, cold allodynia was assessed. The two-way ANOVA of nocifensive score reaction after laying an acetone drop on paws showed a main effect of group [*F*_(1,22)_ = 11.88, *p* < 0.01 and *F*_(1,18)_ = 14.63, *p* < 0.001 in Cohorts 3 and 4, respectively], and a DPI effect in Cohort 4 [*F*_(1,18)_ = 11.26, *p* < 0.01] that was not observed in Cohort 3 [*F*_(1,22)_ = 1.900, *p* > 0.05]. No DPI × group interaction [*F*_(1,22)_ = 1.234, *p* > 0.05 and *F*_(1,18)_ = 3.156, *p* > 0.05 in Cohorts 3 and 4, respectively] was identified. *Post-hoc* analysis showed a significant increase in nocifensive score in QuilA-EAE mice compared to CTL mice at D17 and D30 in Cohort 3 (*p* < 0.05 and *p* < 0.01, respectively), but only at D30 in Cohort 4 (Sidak test, *p* < 0.001) (Figure 3C). These results show the occurrence of cold allodynia in QuilA-EAE mice during the chronic phase of the disease.

#### Evaluation of Orofacial Pain Sensitivity

##### QuilA-EAE Mice Did Not Exhibit Clear Dynamic Mechanical Hyposensitivity

**Figure 4:**
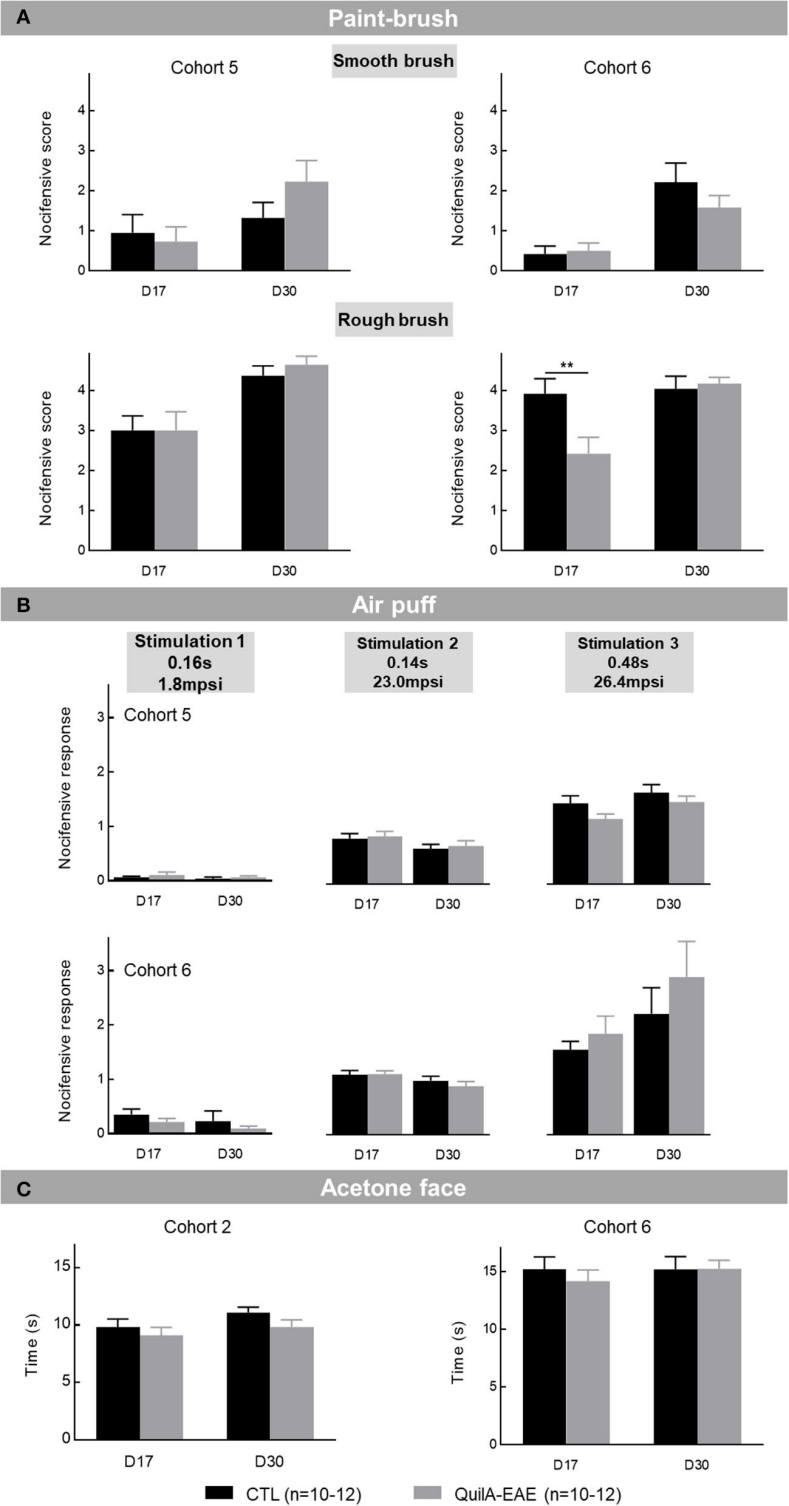
Evaluation of orofacial sensitivity in mice immunized with MOG_35−55_ (QuilA-EAE) and their controls (CTL) at post-induction Day 17 (D17) and D30. **(A)** Mechanical dynamic sensitivity to smooth and rough paintbrushes. Mechanical dynamic sensitivity expressed as the mean number of positive responses ± SEM induced by rubbing the whisker pad with a smooth paintbrush made of marten hairs (top) or a rough paintbrush made of pig bristles (bottom). **(B)** Mechanical static sensitivity to three stimuli of calibrated air puffs (duration and intensity): Stimulation 1 (0.16 s, 1.8 mpsi), Stimulation 2 (0.14 s, 23.0 mpsi), Stimulation 3 (0.48 s, 26.4 mpsi). Mechanical static sensitivity expressed as the mean nociceptive score ± SEM induced by each air puff. **(C)** Cold allodynia expressed as time spent rubbing or scratching face ± SEM for 1 min after acetone drop deposition. Statistical analysis was performed using two-way ANOVA followed by *post-hoc* Sidak test; ^**^p < 0.01 vs. control (shown for Sidak test only).

**Dynamic mechanical sensitivity** was evaluated in Cohorts 5 and 6 using the paintbrush test. The two-way ANOVA of positive responses for smooth brush stimulation showed a main effect of DPI [*F*_(1,20)_ = 4.959, *p* < 0.05 and *F*_(1,22)_ = 33.94, *p* < 0.0001 in Cohorts 5 and 6, respectively]. No group effect [*F*_(1,20)_ = 0.5594, *p* > 0.05 and *F*_(1,22)_ = 0.4740, *p* > 0.05 in Cohorts 5 and 6, respectively] or any DPI × group interaction [*F*_(1,20)_ = 1.844, *p* > 0.05 and *F*_(1,22)_ = 2.060, *p* > 0.05 in Cohorts 5 and 6, respectively] was identified. *Post-hoc* analysis showed no difference between QuilA-EAE and CTL mice at D17 and D30 (Figure 4A, **top**). For rough brush stimulation, the two-way ANOVA analysis showed a main effect of DPI [*F*_(1,20)_ = 25.01, *p* < 0.0001 and *F*_(1,22)_ = 15.57, *p* < 0.001 in Cohorts 5 and 6, respectively]. In Cohort 5, neither a group effect [*F*_(1,20)_ = 0.1373, *p* > 0.05] nor any DPI × group interaction [*F*_(1,20)_ = 0.1650, *p* > 0.05] was identified, whereas in Cohort 6, DPI × group interaction [*F*_(1,22)_ = 11.69, *p* < 0.01] was identified but had no effect of group [*F*_(1,22)_ = 3.020, *p* > 0.05]. *Post-hoc* analysis in Cohort 6 showed a significant decrease in the number of positive responses only at D17 (Sidak test, *p* < 0.01), which was not observed in Cohort 5 (Figure 4A, **bottom**). These results, showing some discrepancies between cohorts, do not unequivocally show any transitory orofacial mechanical hyposensitivity during QuilA-EAE disease onset.

##### QuilA-EAE Mice Exhibited Normal Static Mechanical Sensitivity

**Static mechanical sensitivity** was assessed by air the puff test. In Cohorts 5 and 6, the two-way ANOVA showed a significant main effect of air puffs [*F*_(2,120)_ = 51.38, *p* < 0.0001 and *F*_(2,132)_ = 262.2, *p* < 0.0001 in Cohorts 5 and 6, respectively]. In Cohort 5, no group effect [*F*_(3,120)_ = 0.6541, *p* > 0.05] and air puffs × group interaction [*F*_(6,120)_ = 2.147, *p* = 0.0529] was identified. In Cohort 6, an air puffs × group interaction [*F*_(6,132)_ = 3.103, *p* < 0.001] was identified but had no group effect [*F*_(3,132)_ = 0.4766, *p* > 0.05]. In both Cohorts 5 and 6, *post-hoc* analysis showed no difference in the number of nocifensive responses between EAE and CTL mice at D17 and D30 with whatever stimulation used (Figure 4B). These results show no facial static mechanical sensitivity alteration in QuilA-EAE mice.

##### QuilA-EAE Mice Exhibited No Facial Cold Allodynia

**Facial cold allodynia** was evaluated to complete orofacial sensitivity characterization. In Cohorts 2 and 6, the two-way ANOVA failed to show any effect of DPI [*F*_(1,22)_ = 2.979, *p* > 0.05 and *F*_(1,22)_ = 0.5853, *p* > 0.05 in Cohorts 2 and 6, respectively], group [*F*_(1,22)_ = 2.087, *p* > 0.05 and *F*_(1,22)_ = 0.1662, *p* > 0.05 in Cohorts 2 and 6, respectively], and DPI × group interaction [*F*_(1,22)_ = 0.2039, *p* = 0.6829 and *F*_(1,22)_ = 0.6694, *p* = 0.5818 in Cohorts 2 and 6, respectively] (Figure 4C). These results show no facial cold allodynia development in QuilA-EAE mice.

#### Cognitive Evaluation

##### QuilA-EAE Mice Exhibited No Clear Social Interaction Deficit

**Figure 5:**
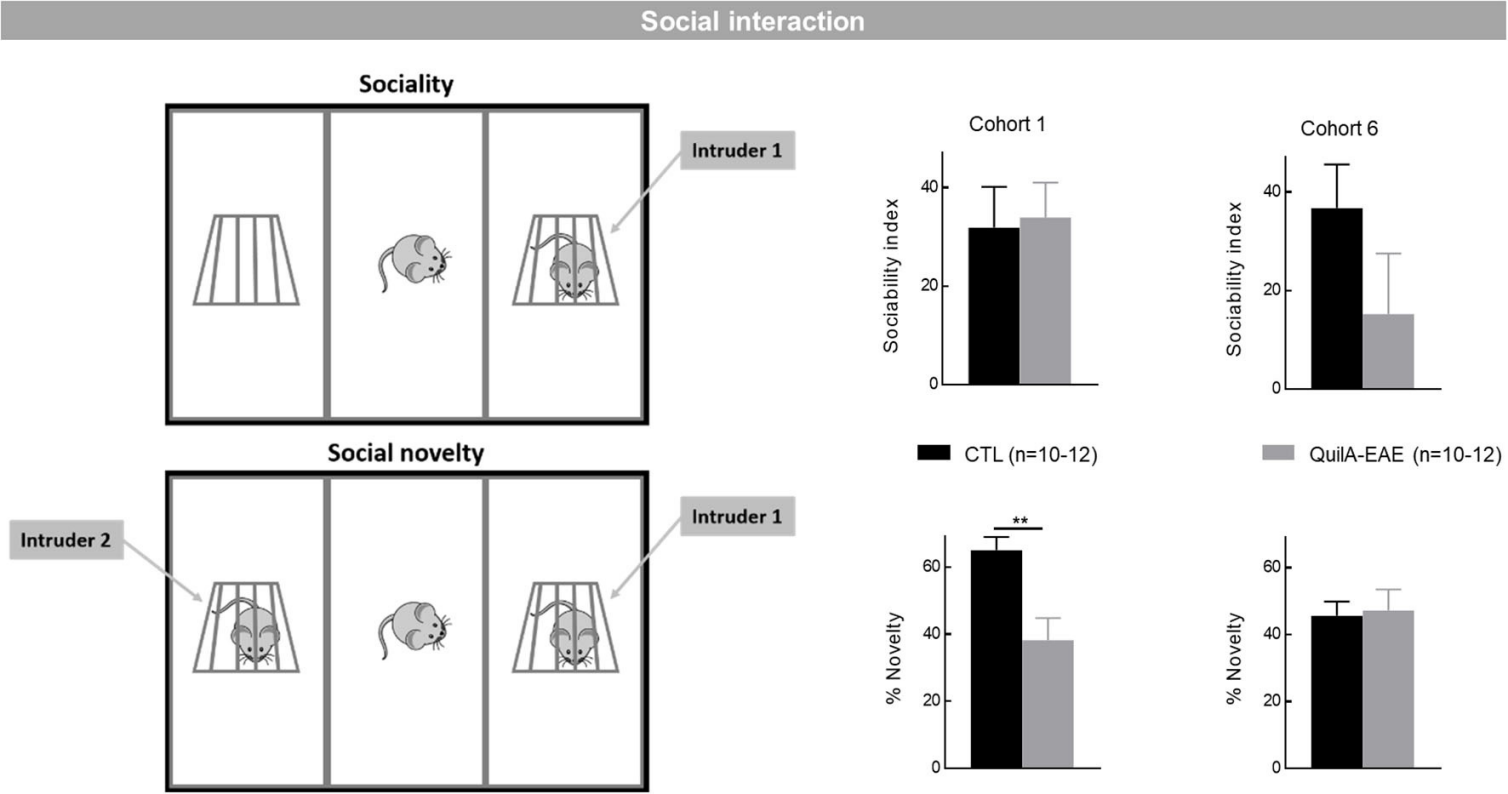
Evaluation of cognitive behaviors using the three-chamber sociability and social novelty test in mice immunized with MOG_35−55_ (QuilA-EAE) and their controls (CTL) at post-induction Day 30. Left: Schematic representation of the three-chamber sociability and social novelty test. Top: the T1 session when an unfamiliar adult WT female mouse was placed in one of the wire cups for the evaluation of sociability. Bottom: the T2 session where the first intruder is still posted in its wire cup but another unfamiliar adult WT female mouse was placed in the second cup for the evaluation of preference for social novelty. Top right: Sociability evaluated using the sociability index (exploration time of unfamiliar mice – exploration time of empty cup / total exploration time of both cups) ± SEM over the T1 5 min session. Bottom right: Social novelty evaluated using the percentage of novelty [(exploration time of unfamiliar mice/total exploration time of both mice) × 100] ± SEM for the T2 5 min session. Statistical analysis was performed using Student *t*-test, ^**^p < 0.01 vs. control.

**Sociability and social novelty preference** were evaluated in Cohorts 1 and 6 at D30 during the chronic phase of the disease. The statistical analysis showed no difference in the sociability index between QuilA-EAE and CTL mice in either cohort (Student *t*-test, *p* > 0.05) (Figure 5, **top**). When the percentage of novelty was analyzed, in Cohort 1, a significant decrease was observed in QuilA-EAE mice compared to CTL mice (Student *t*-test, *p* < 0.01), whereas no difference between groups was shown in Cohort 6 (Student *t*-test, *p* > 0.05 for both) (Figure 5, **bottom**). From these results, QuilA-EAE mice did not exhibit any sociability impairment, whereas social novelty preference could be altered but with some discrepancies between cohorts.

##### QuilA-EAE Mice Exhibit Neither Episodic Memory Impairment Nor Working Memory Deficit

**Episodic memory** was assessed in Cohorts 1 and 2 using the novel object recognition test. Statistical analysis showed no difference in the percentage of recognition and index of recognition between QuilA-EAE and CTL mice (Student *t*-test, *p* > 0.05) (Table 3, **Object recognition test**).

**Table 3:** Summary table of cognitive tests performed in mice immunized with MOG_35−55_ (QuilA-EAE) and their controls (CTL) during the chronic phase of the disease (D30).

**Object recognition test** **% of recognition**		**Y-maze % of alternation**		**Elevated plus maze, Time in open arms (s)**		**Marble burying test, Number of marbles buried**		**Open field test, Time spent in central area (s)**	
**Cohort 1 (*****n*** **= 7)**		**Cohort 2 (*****n*** **= 12)**		**Cohort 2 (*****n*** **= 12)**		**Cohort 2 (*****n*** **= 12)**		**Cohort 3 (*****n*** **= 12)**	
CTL 67.43 ± 4.87 EAE 59.86 ± 0.94	NS	CTL 56.41 ± 2.50 EAE 55.93 ± 2.35	NS	CTL 17.53 ± 3.60 EAE 23.83 ± 4.58	NS	CTL 9.08 ± 1.52 EAE 8.83 ± 1.20	NS	CTL 94.38 ± 14.8 EAE 78.68 ± 9.02	NS
**Cohort 2 (*****n*** **= 8–12)**		**Cohort 3 (*****n*** **= 12)**		**Cohort 3 (*****n*** **= 12)**		**Cohort 3 (*****n*** **= 12)**		**Cohort 4 (*****n*** **= 12)**	
CTL 67.64 ± 2.18 EAE 62.97 ± 2.23	NS	CTL 56.50 ± 2.59 EAE 55.90 ± 2.47	NS	CTL 25.29 ± 7.11 EAE 17.52 ± 5.08	NS	CTL 6.58 ± 1.19 EAE 5.83 ± 0.83	NS	CTL 78.92 ± 11.4 EAE 74.38 ± 10.46	NS

*Episodic memory was assessed using object recognition. The percentage of recognition during a 5 min exploration session was used as episodic memory index*.

*Working memory was evaluated using the Y-maze test. Percentage alternation is an index of working memory and was calculated for each mouse in a 10 min session*.

*Anxiety was assessed using three different tests: (i) the elevated plus maze for which we evaluated the time spent in open arms measured during a 10 min session, (ii) the open field test for which we evaluated the time spent in the central area ± SEM in the overall 15 min session, and (iii) the marble burying test for which the number buried was determined after a 30 min session*.

*All test results are means ± SEM. Statistical analysis was performed using Student t-test except for the EPM results of Cohorts 2 and 3 where assumptions of normality were not met and the object recognition results of Cohort 1 where the assumption of equality of variance was not met. For these data, a Mann-Whitney test was performed. NS, non-significant*.

**Working memory** was evaluated using the Y-maze in Cohorts 3 and 6. Statistical analysis showed no difference in the percentage of alternation comparing QuilA-EAE to CTL mice (Mann-Whitney, *p* > 0.05) (Table 3, **Y-maze**).

##### QuilA-EAE Mice Exhibited No Anxiety-Related Behaviors

**Anxiety-related behaviors** were assessed using three different tests. First, using the EPM test in Cohorts 3 and 2, statistical analyses showed no difference in time spent in open arms comparing QuilA-EAE to CTL mice (Mann-Whitney, *p* >0.05) (Table 3, **EPM test**). Second, using the marble burying test in the same cohorts, statistical analysis showed no difference in number of marbles buried between QuilA-EAE and CTL mice (Student *t*-test, *p* > 0.05) (Table 3, **marble burying test**). Third, using the open-field test in Cohorts 3 and 4, statistical analysis showed no difference in the time spent in the central area between QuilA-EAE and CTL mice (Student *t*-test, *p* > 0.05) (Table 3, **open field test**). All these data converge to indicate that the QuilA-EAE mice exhibited no anxiety-related behaviors.

### Pharmacological Impact on Somatic Sensitivity

#### Dose-Dependent Analgesic Effect of Pregabalin in QuilA-EAE and CTL Mice, Assessed on Mechanical Sensitivity

**Figure 6:**
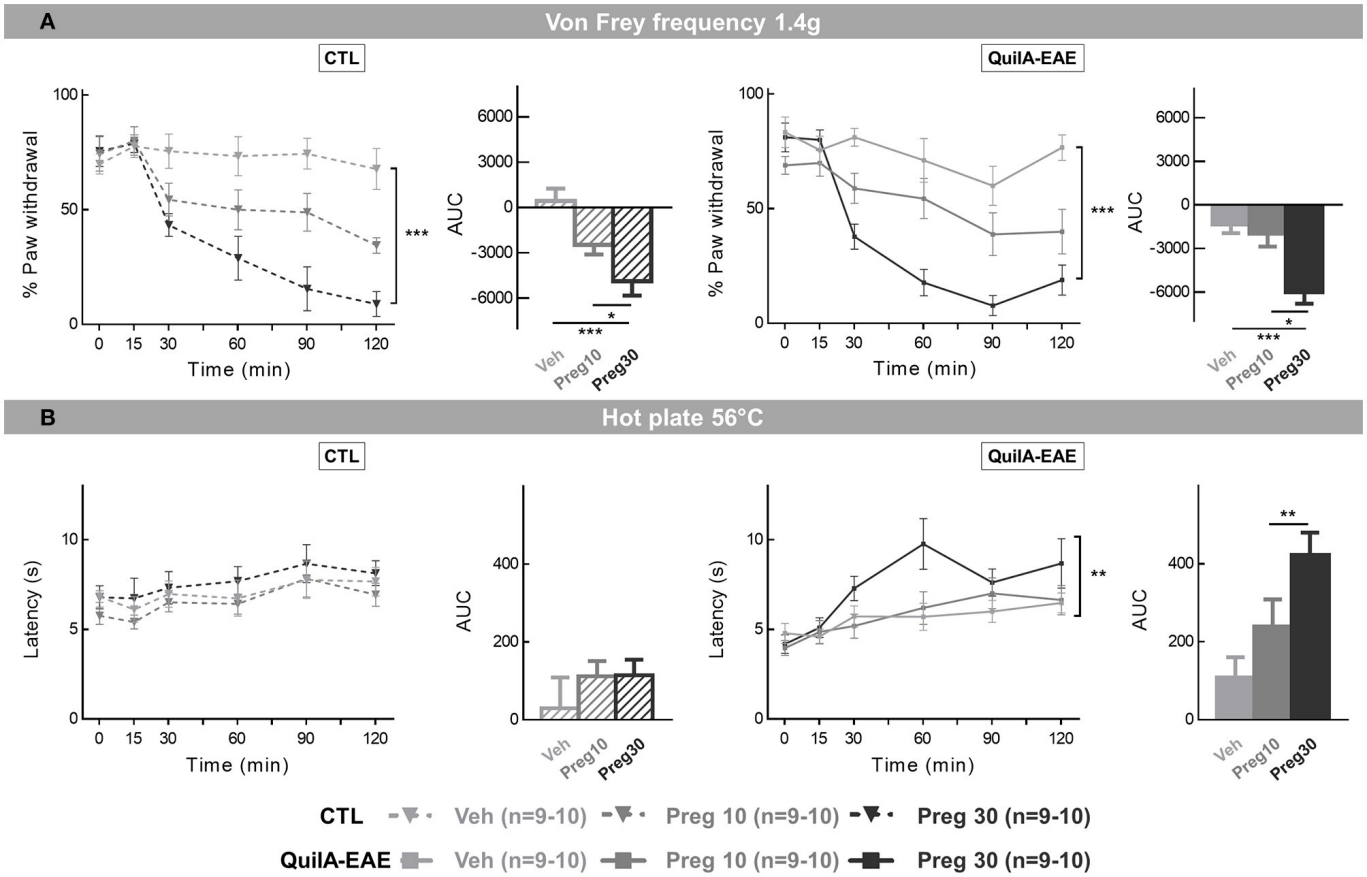
Pharmacological evaluation of pregabalin effects on mechanical sensitivity and heat hyperalgesia in mice immunized with MOG_35−55_ (QuilA-EAE) and their controls (CTL) during the chronic phase of the disease. Mice received a single intraperitoneal (i.p.) bolus dose of pregabalin at 10 or 30 mg/kg or vehicle (NaCl sterile for injection). **(A)** Mechanical sensitivity assessed by the von Frey frequency method using calibrated 1.4 g von Frey hair filaments. Measurements were made before injection and for 2 h after injection and expressed as the mean percentage of paw withdrawal ± SEM. Results are represented as time course curves for each group and as areas under the curve (AUC) calculated for each group and represented by a bar chart. Data for CTL mice are given on the left and data of QuilA-EAE mice on the right. **(B)** Heat hyperalgesia evaluated by a hotplate test at 56°C. Measurements were made before injection and 2 h after injection and expressed as the mean latency of the first nocifensive behavior ± SEM. Results are represented as a time course curve for each group and as area under the curve (AUC) calculated for each group and represented by a bar chart. Data of CTL mice are given on the left, data of QuilA-EAE mice on the right. For AUC, statistical analysis was performed using one-way ANOVA followed by *post-hoc* Tukey's test; ^*^p < 0.05, ^**^p < 0.01, ^***^p < 0.001.

Pregabalin effect was evaluated on mechanical hyperalgesia at D30 in EAE mice after hypersensitivity was fully developed. Over a 2 h follow-up, the effect of vehicle (NaCl) or pregabalin (10 and 30 mg/kg, i.p. injection) was evaluated by the percentage of paw withdrawal evoked by a von Frey hair filament (1.4 g) in QuilA-EAE and CTL mice. The one-way ANOVA of the area under the curves obtained for each group and treatment showed a significant main effect of treatments [*F*_(2,24)_ = 10.75, *p* < 0.001 and *F*_(2,24)_ = 11.42, *p* < 0.001 in CTL and QuilA-EAE groups, respectively]. *Post-hoc* analysis showed a significant decrease in the percentage of paw withdrawal for CTL mice treated with pregabalin 10 and 30 mg/kg (Sidak test, *p* < 0.01 and *p* < 0.001 for Preg10 and Preg30 compared to vehicle, respectively) (Figure 6A, **left**). A significant decrease in the percentage of paw withdrawal was observed only in QuilA-EAE mice treated with pregabalin 30 mg/kg (Tukey's test, *p* < 0.001 *vs*. vehicle) (Figure 6A, **right**). Similar data were analyzed for a.4 g von Frey hair filament (Table 4). The one-way ANOVA analysis of AUC showed a significant main effect of treatment [*F*_(2,24)_ = 4.779, *p* < 0.05 and *F*_(2,24)_ = 4.010, *p* < 0.05 in CTL and QuilA-EAE groups, respectively]. *Post-hoc* analysis showed a significant decrease in the percentage of paw withdrawal for CTL and QuilA-EAE mice treated with pregabalin 30 mg/kg (Tukey's test, *p* < 0.05 vs. vehicle). These results show an analgesic effect of pregabalin on mechanical sensitivity in CTL and QuilA-EAE mice.

**Table 4:** Summary table of pharmacological effect of pregabalin on mechanical and thermal sensitivity in mice immunized with MOG_35−55_ (QuilA-EAE) and their controls (CTL) during the chronic phase of the disease.

**AUC Von Frey 0.4 g**	**Group**	**Vehicle**	**Pregabalin 10 mg/kg**	**Pregabalin 30 mg/kg**
	CTL	125 ± 409.1	−1,325 ± 769.1	−3,258 ± 1025^*^
	EAE	−1,442 ± 896.5	−2,667 ± 844.6	−4,692 ± 706.1^*^

*Mice received a single intraperitoneal (i.p.) bolus dose of pregabalin at 10 or 30 mg/kg or vehicle (NaCl sterile for injection)*.

*Comparison of area under the curve (AUC) of the percentage of paw withdrawal assessed by von Frey frequency method using calibrated 0.4 g and 1.4 g filaments and the mean latency of the first nocifensive behavior observed on a hotplate at 56°C*.

*Statistical analysis was performed using one-way ANOVA followed by post-hoc Tukey's test*;

* *p < 0.05 vs. vehicle*.

Due to the important effect of pregabalin observed in CTL mice, we decide to run a control experiment to eliminate a possible locomotor confounding effect of the drug. Pregabalin effect was evaluated on spontaneous locomotion using the open-field test in wild-type female mice. Over a 2 h follow-up, the effect of vehicle (NaCl) or pregabalin (10 and 30 mg/kg, i.p. injection) was evaluated by the total distance traveled. The one-way ANOVA of the area under the curves obtained for each treatment showed no main effect of treatments [*F*_(2,25)_ = 1.202, *p* > 0.05] (Supplementary Figure S2).

#### Dose-Dependent Analgesic Effect of Pregabalin in QuilA-EAE and CTL Mice, Assessed on Heat Hyperalgesia

To complete this pharmacological characterization, the potential analgesic effect of pregabalin on heat hyperalgesia was assessed. Over a 2 h follow-up, the effect of vehicle (NaCl) or pregabalin (10 and 30 mg/kg, i.p. injection) was evaluated using the hotplate at 56°C. The one-way ANOVA of latency of first nocifensive response showed a main effect of treatment in the QuilA-EAE group [*F*_(2,27)_ = 6.777, *p* < 0.01] but not the CTL group [*F*_(2,27)_ = 0.7424, *p* > 0.05]. *Post-hoc* analysis showed a significant increase in latency in QuilA-EAE mice treated with pregabalin at 30 mg/kg (Tukey's test, *p* < 0.01 vs. vehicle) (Figure 6B, **right side**). These results show an analgesic effect of the higher dose of pregabalin on heat hyperalgesia in QuilA-EAE but not CTL mice.

## Discussion

Briefly, our results show that QuilA-EAE mice developed both a severe and lasting specific mechanical allodynia, fully developed at D30 after disease induction, heat hyperalgesia, and cold allodynia, with no motor impairment. They did not present orofacial sensitivity impairment. Cognition and anxiety did not seem significantly modified. Finally, an antineuropathic pain treatment (pregabalin) had a clear analgesic effect. This MS model thus seems well-suited for exploring neuropathic pain due to inflammatory CNS lesions.

### Reproducibility of the QuilA-EAE Mouse Model

The first description of an EAE model in which CFA was replaced by Quil A was published in 2007 (27), but this model was then largely forgotten until the work of Khan et al. (15) and its recent use by a Brazilian team (20). Before reporting our new results with this model, we set out to compare our results replicating those already reported in the literature. First, we successfully replicated this model without major difficulty according to the protocols described (15, 27). We note that the young age (6 weeks) of female mice at immunization is a very important parameter which have to be respected, and that for an unknown reason, some cohorts of mice failed to be correctly immunized (in our experiments between 15 and 20%). This variability of immunization has already been described in more classical EAE models (28, 30). Second, because the model described by Peiris et al. is slightly different from those described by Khan et al., it was important to position our data relative to these two descriptions. Immunization protocols differed slightly according to the MOG_35−55_ peptides used. Particularly, those commercialized by Mimotopes Pty Ltd. in Peiris et al. vs. those commercialized by Sigma Aldrich in the later studies. The adjuvant doses used also differed. The first study used 15 μg Quil A and 200 ng PTX, whereas the second used 45 μg Quil A and 250 ng PTX. In this study, we used the immunization protocol described by Khan et al. (15). For the time course of the EAE score, our data were closer to those of Khan et al. (15) than to those of Peiris et al. (27). Both papers described a first clinical episode with a mean clinical score peaking between 1.5 and 2, followed by recovery and subsequent relapses and recoveries. In our hands, EAE mice reproduced alternating relapses and recoveries. However, the first clinical episode occurred around D16 as also in Khan et al. vs. around D12 in Peiris et al. Other slight differences were observed concerning the recovery, which occurred at 4–6 days after the first clinical episode and persisted for 9–10 days in Khan et al., like here. On the other hand, in Peiris et al., the recovery occurred at 5–6 days after the first clinical episode and persisted for 10–12 days. Another discrepancy is that no clinical score was observed during recovery in Peiris et al. while a mean clinical score of 0.8 was always observed in the later studies. Finally, while Peiris et al. found that relapses were always less severe than the first clinical episode, we, like Khan et al., observed no such phenomenon at least for the first three relapses observed.

Regarding mechanical allodynia evaluated using the von Frey test, we confirmed the data of Khan et al. and found a mechanical allodynia fully developed at D30 (appearing around D23–D26) after disease onset and motor impairment in QuilA-EAE mice.

### Extended Characterization of Sensitive Behaviors

In most studies evaluating somatic sensitive behaviors in EAE mice (whatever the variant used), mechanical allodynia was always assessed, whereas heat hyperalgesia was less often studied. Studies assessing the latter parameter showed divergent results for the acute EAE phase of the disease, with some demonstrating heat hypoalgesia (29, 44, 45) and others heat hyperalgesia (46–48). Data related to the chronic phase of the disease were more closely consistent with all the studies showing heat hyperalgesia in EAE mice. The results obtained in our study showed for the first time that QuilA-EAE mice developed heat hyperalgesia during the chronic phase of the disease. Concerning cold allodynia, in our study, it occurred in the early phase of the disease. This observation in QuilA-EAE is similar to those made in different variants of EAE in which cold allodynia has always been described as an early symptom that worsens during the disease course (20, 29, 46, 49, 50).

Concerning orofacial sensitivity in the QuilA-EAE model, unlike one publication using the EAE model with CFA showing increased sensitivity to air puffs applied to the whisker pad (38), we observed no major difference in sensitivity to this kind of stimulation. In fact, we observed no difference between CTL and QuilA-EAE mice for air puff stimulation, and only a transitory hyposensitivity to the rough paintbrush. This last result invites caution because it was observed in one cohort but not replicated in a second one. Finally, for orofacial cold stimulation using acetone drops, we did not observe any difference between groups. To our knowledge, we are the first to evaluate this effect in an EAE mouse model. In summary, our findings suggest the probable absence of frequent orofacial mechanical and cold allodynia in QuilA-EAE mice. In CFA-EAE mice, the increased orofacial sensitivity has been associated with (i) immune cell infiltration and glial cell (satellites glia or astrocytes/microglia) activation in the trigeminal ganglia and the trigeminal brainstem complex, and (ii) demyelination in the peripheral myelin transition zone and the intrapontine trigeminal sensory root and spinal trigeminal tract (38). Conversely, in the QuilA-EAE mice neuropathological data are mostly restricted to the spinal cord. Brain data are very scant, provided only in the first article of Khan et al. where a decrease in myelin basic protein (MBP) and an increase in glial fibrillary acidic protein (GFAP) and ionized calcium-binding adapter molecule 1 (Iba1) immunostainings are described in lateral corpus callosum (15). This publication also described alteration in the hippocampus but without illustration and quantification. Future neuropathological evaluations of trigeminal ganglia or brainstem in QuilA-EAE mice are then of huge interest to understand the mechanistic differences between the two EAE variants. In the same way, a detailed assessment of encephalic damage (especially at the level of limbic system) would be of particular interest in view of our assessment of the interfering symptoms presented below.

Finally, we showed that pregabalin could correct both mechanical allodynia and thermal hyperalgesia in QuilA-EAE mice. This pharmacological validation cannot ignore the findings of a recent meta-analysis showing that pregabalin effect in preclinical models was not always predictive of clinical results. Notably, this meta-analysis highlights that in experiments testing acute nociceptive pain etiologies, pregabalin demonstrated robust pooled effect sizes, whereas its clinical efficacy in acute and chronic non-neuropathic pain was practically nil (51).

### Extending the Behavioral Evaluation to Cover Interfering Symptoms Observed in MS Patients

In a disease as composite as MS, evaluating an animal model focusing on a single kind of disability (motor, sensitive, or cognitive) is probably too restrictive, and a broader evaluation is probably needed for successful translational research. This is particularly true in that pain shows greater interference in MS, notably with fatigue, mood disturbances (depression and anxiety), and sometimes cognitive impairment (4, 6, 24). It is thus imperative to characterize a new model with behavioral methods that are sensitive to the array of impairments arising in MS.

#### Evaluation of Motor Dysfunctions in QuilA-EAE Mice

Accurately defining motor defects from slight to major dysfunction is a key difficulty not only in MS research but also in other neurologic diseases with different levels of motor dysfunctions according to the disease stage, such as stroke or Parkinson's disease (52, 53). The most common tests used to assess motor function in mice are spontaneous locomotor activity in the open field and coordination in the rotarod test. However, both tests often lack the sensitivity needed to detect subtle alterations. In this variant of the EAE model in which motor dysfunctions are voluntary restricted, it is furthermore important to be able to detect these subtle alterations. As described by Khan et al. (15), we confirmed the absence of severe motor dysfunction with a mean EAE clinical score never exceeding 2 (limp tail with unilateral partial hindlimb paralysis), strengthened by results obtained in more dedicated tests: the open field, rotarod, and grip strength tests. Strikingly, our results obtained in the open field and the rotarod tests were in agreement with data obtained by Dalenogare et al. (20). Finally, weak impairments of fine motor coordination were observed only using the grid test, which enabled us to demonstrate and quantify this symptom in QuilA-EAE mice. The grid test is known to highlight subtle ataxic behaviors in mice, often related to cerebellar dysfunctions (54).

#### Evaluation of Anxiety-Related Behaviors in QuilA-EAE Mice

Anxiety disorders are found in 37.5% of MS patients (55). Until now, anxiety-like behaviors were only described in the CFA EAE mouse model. These experimentations showed a reduced exploratory behavior using the light/dark box test (56, 57), the elevated plus maze (58), and open field test (56, 58). In all these studies, behavioral experiments were performed during the pre-symptomatic phase of the disease before the appearance of motor defects. To our knowledge, we are the first to evaluate these behaviors in an QuilA-EAE model. Our data using the elevated plus maze, marble burying, and the open field test showed no anxiety-related behaviors in QuilA-EAE mice. The discrepancy between our results and those already described in the literature might be due to the difference between CFA and QuilA-EAE models or the difference in the timing of the behavioral evaluation (presymptomatic vs. symptomatic phase of the disease). Further experiments are now needed to conclude.

#### Evaluation of Cognitive Behaviors in QuilA-EAE Mice

Some 45–60% of MS patients show cognitive impairment and several domains are affected, such as long-term and working memory, executive function, attention, and speed of information processing (22, 23). Social impairment and social cognition deficits have also been observed in MS (59).

Data on cognitive function in EAE mice are scant. The first studies included that of Olechowski, showing defects in working memory in a CFA EAE model using the novel object recognition test (60). De Bruin et al. showed a decrease in social recognition in the QuilA-EAE model induced in SJL mice using the social preference and social novelty test (61). Recently, a deficit of hippocampal memory was demonstrated in a CFA EAE model using a Barnes maze or a dedicated contextual fear conditioning procedure (62, 63) or in a chronic-relapsing EAE model using the hole-board test (64). In line with the literature, the data obtained in our study confirmed that if cognition defects do occur in QuilA-EAE mice, then they are slight. In fact, in only one cohort did we find a decrease in social recognition using the social preference and social novelty test. None of the other cognitive functions evaluated (working and episodic memories, anxiety-related behaviors) were affected.

The very limited occurrence of anxiety-related behaviors or cognitive dysfunctions in our study can be seen as a limitation of the QuilA-EAE model. However, before drawing conclusions, some aspects have to be taken into account. First, in our study, interfering symptoms were evaluated only at D30. It is thus still possible that some symptoms could arise earlier or later in the disease time course. Secondly, we did not evaluate depression-like behaviors in our model. Thirdly, regarding cognitive symptoms, in patients, information processing speed reduction is usually the first cognitive alteration and concerns only 20–25% of the patients at disease onset (65). It is thus probably difficult to explore such minor alterations in animal models, although they may be present. Finally, as in our experimental set up, QuilA-EAE and controls mice are bred together, a possible explanation of the moderate behavioral phenotype observed is a possible “fecal transplants” through coprophagia. Given that the vast majority of fecal transfer experiments realized in EAE mice described in literature use germ-free mice (66) or treatment with antibiotic cocktails during few days to weeks (67, 68) to ensure extensive microbiota changes, we assume that the probability of possible 'fecal transplants' through coprophagia is very low but still possible.

### Potential “Bottom-Up” Translational Value of Our Study

For some years, there has been a disappointing lack of translational progress in the pain field. The accumulating basic scientific knowledge obtained using animal models, though promising, has so far failed to produce new drugs that are efficacious in human patients. Although the reasons for these failures are probably numerous, some commentators have posited that inadequate design, conduct, and reporting of preclinical experiments may explain them (69–71). However, recent studies suggest that factors other than quality of preclinical studies may play a greater role in explaining discrepancies between effects observed in non-human animals and those in patients. Notably, latent environmental factors, such as housing, diet, and experimenter sex may affect stress levels in the preclinical testing environment, influencing both behavioral and non-behavioral outcomes (51, 70, 72). One proposed way to minimize this caveat is environmental heterogenization. In other words, increasing the within-experiment variation which could be achieved by applying systematic variation of experimental conditions or supporting the reproduction of the experiments in at least two different laboratories. Though appealing, both solutions have limits: the first presupposes which environmental factor truly matters, and the second multiplies the number of animals needed. In this study, we propose an in-between solution where the environmental factors of our own animal facility were evaluated by assessing each test in two different cohorts. The idea was that the behavioral defects replicated in both cohorts were highly relevant to the model and not an artifact due to environmental factors. Although the conditions of our animal facility were highly controlled, slight variations were still possible (barometric pressure, presence of other mice in the stability room, difference in the amount of handling before testing, etc.) that might explain the presence in some tests of differences observed in one cohort but not replicated in the second. This suggests a higher sensitivity to environmental modifications for these tests, namely, grid, social novelty, and paintbrush tests. Very strikingly, the discrepancy between the two cohorts seems not always imputable to difference in EAE mice behaviors. Notably, in social novelty, the absence of difference in Cohort 6 seemed more related to a decrease in social performance in CTL mice rather than in QuilA-EAE mice.

## Conclusion

Taken together, in this study we successfully replicated the QuilA-EAE model described by Khan et al. (15) with a high degree of similitude. We further characterized this model as regards its sensitive presentation and demonstrated that these QuilA-EAE mice associated mechanical allodynia with thermal hyperalgesia and cold allodynia after somatic stimulations. For orofacial stimulations, data suggested no major sensitive defect in QuilA-EAE for this kind of stimulation. We then broadened the characterization to cover other kinds of symptoms and showed that subtle motor defects in this model could be assessed not only by the EAE score but also using the grid test. However, these motor defects were far too slight to elicit abnormal results in the open field, the rotarod, or the grip strength test. Finally, we show that QuilA-EAE mice could be susceptible to social memory defects but did not exhibit episodic or working memory defects or anxiety-related behaviors. All these data confirm that this QuilA-EAE model represents a useful new model of MS. It now needs further neuropathological characterization before it can be used to identify new therapeutic agents dedicated to sensitive defects in MS.

## Data Availability Statement

The raw data supporting the conclusions of this article will be made available by the authors, without undue reservation.

## Ethics Statement

The animal study was reviewed and approved by the agreement of local Ethic Committee and the french Ministère de l'Enseignement Supérieur et de la Recherche (CEMEA-Auvergne APAFIS-4306).

## Author Contributions

AD performed the experiments, derived the models, analyzed, the data and wrote the first draft of the manuscript. BS contributed to data or analysis tools, discussed the results, and contributed to the final manuscript. FG, XM, and LD discussed the results and contributed to the final manuscript. AE supervised the project, discussed the results and contributed to the final manuscript. MB designed and directed the project, contributed to data or analysis tools, and participated to all the step of the manuscript preparation. All authors contributed to the article and approved the submitted version.

## Funding

This work was supported by the Ministère français de l'Enseignement supérieur et de la Recherche (AD Ph.D. fellowship) and French government IDEX-ISITE initiative (grant number 16-IDEX-0001-CAP 20-25) of the University of Clermont Auvergne. It was specifically funded by grants from ARSEP Foundation (AAP 2014).

## Conflict of Interest

The authors declare that the research was conducted in the absence of any commercial or financial relationships that could be construed as a potential conflict of interest.

## Publisher's Note

All claims expressed in this article are solely those of the authors and do not necessarily represent those of their affiliated organizations, or those of the publisher, the editors and the reviewers. Any product that may be evaluated in this article, or claim that may be made by its manufacturer, is not guaranteed or endorsed by the publisher.

## Supplementary Material

Supplementary Figure S1 Determination of reproducibility of air puff for each type of stimulation. Left: raw data of 60 stimulations mimicking the experimental procedure. Right: mean and confidence interval (CI) for each type of stimulation. The characterization and the reproducibility of the air puff were measured for three different stimulations (1–3) as described. Stimulation recording systems were composed of a force transducer MLTF500/ST (ADInstruments Ltd, Paris, France) connected to a PowerLab 26T USB data acquisition device (ADInstruments Ltd., Paris, France) coupled to the Labchart 7 (ADInstruments Ltd, Paris, France). To register the air puff exerted by each stimulation, a square card measuring 2.6 cm^2^ was fixed on the force transducer. Air puffs were recorded in a range of 1 mV, low pass 50 Hz, with a trigger fixed at 2 μV. Response to stimulation was recorded for 100 ms before and 1 s after, with a sample rate of 10 kHz. For each stimulation (1–3), 60 air puffs were applied to the card and data recorded with Labchart. To determine the characteristic of the stimulations (1–3), the curves illustrating air puff variations in volts were analyzed using the Labchart to measure peak duration (s) and max intensity (μV). Graphical representations were obtained after data were extracted with MATLAB (MathWorks, Natick, USA) and mean confidence intervals for each stimulation were calculated and converted to pounds per square inch (psi).

Supplementary Figure S2 Pharmacological evaluation of pregabalin effects on locomotor activity using the open-field test in wild-type (WT) female mice. Evaluation of spontaneous locomotor activity using the open-field test. Locomotion was evaluated using the total distance traveled ± SEM for the overall 120 min session. For AUC, statistical analysis was performed using one-way ANOVA followed by *post-hoc* Tukey's test; NS.
