# Supplementary figures and images for: In-Depth Characterization of Somatic and Orofacial Sensitive Dysfunctions and Interfering-Symptoms in a Relapsing-Remitting Experimental Autoimmune Encephalomyelitis Mouse Model
Source: Front Neurol. 2022 Jan 17;12:789432. doi: 10.3389/fneur.2021.789432 (PMC8801881; doi:10.3389/fneur.2021.789432)

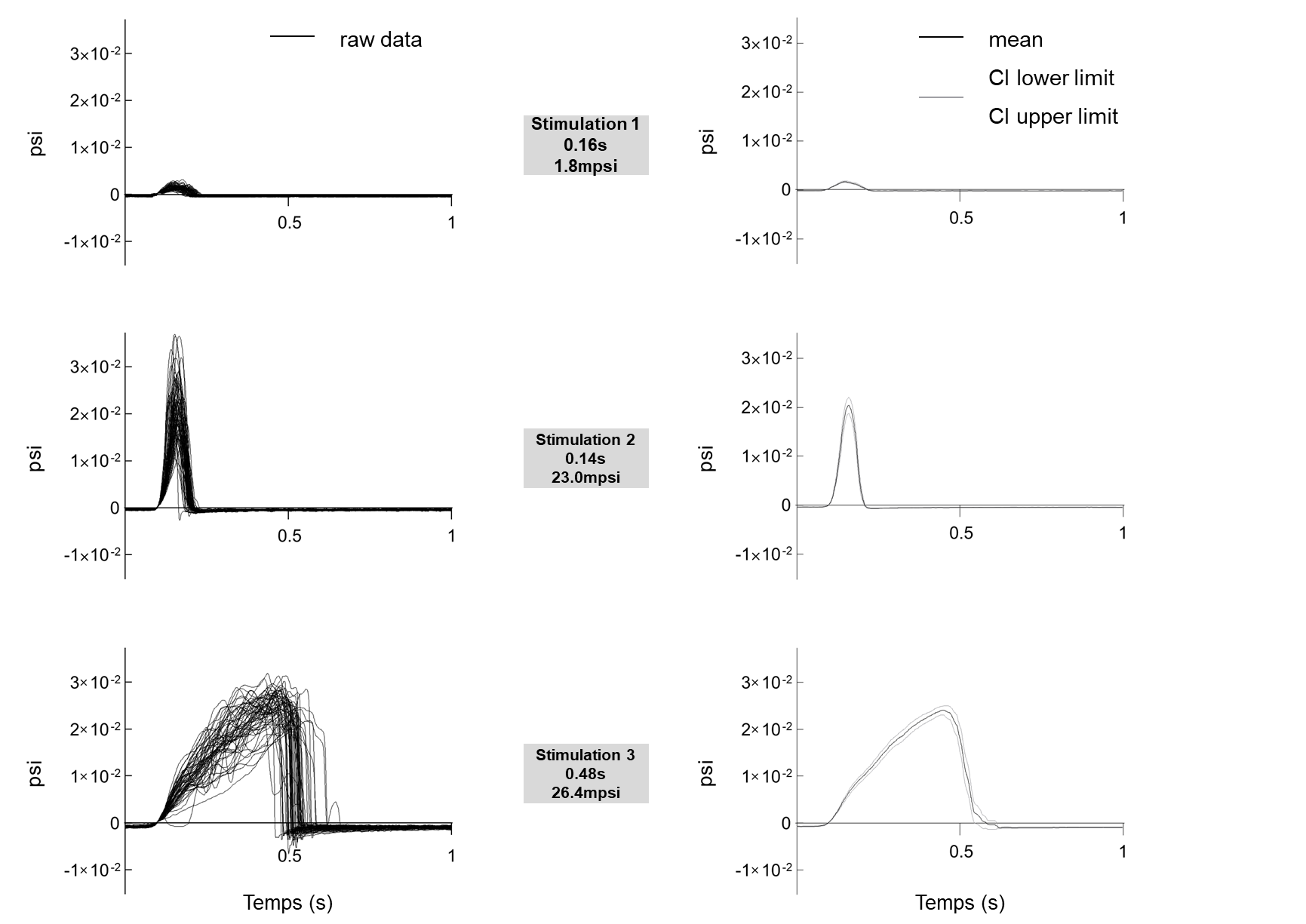

Supplement: Supplementary Figure S1 — Determination of reproducibility of air puff for each type of stimulation. Left: raw data of 60 stimulations mimicking the experimental procedure. Right: mean and confidence interval (CI) for each type of stimulation. The characterization and the reproducibility of the air puff were measured for three different stimulations (1–3) as described. Stimulation recording systems were composed of a force transducer MLTF500/ST (ADInstruments Ltd, Paris, France) connected to a PowerLab 26T USB data acquisition device (ADInstruments Ltd., Paris, France) coupled to the Labchart 7 (ADInstruments Ltd, Paris, France). To register the air puff exerted by each stimulation, a square card measuring 2.6 cm2 was fixed on the force transducer. Air puffs were recorded in a range of 1 mV, low pass 50 Hz, with a trigger fixed at 2 μV. Response to stimulation was recorded for 100 ms before and 1 s after, with a sample rate of 10 kHz. For each stimulation (1–3), 60 air puffs were applied to the card and data recorded with Labchart. To determine the characteristic of the stimulations (1–3), the curves illustrating air puff variations in volts were analyzed using the Labchart to measure peak duration (s) and max intensity (μV). Graphical representations were obtained after data were extracted with MATLAB (MathWorks, Natick, USA) and mean confidence intervals for each stimulation were calculated and converted to pounds per square inch (psi). [file Image_1.TIF]

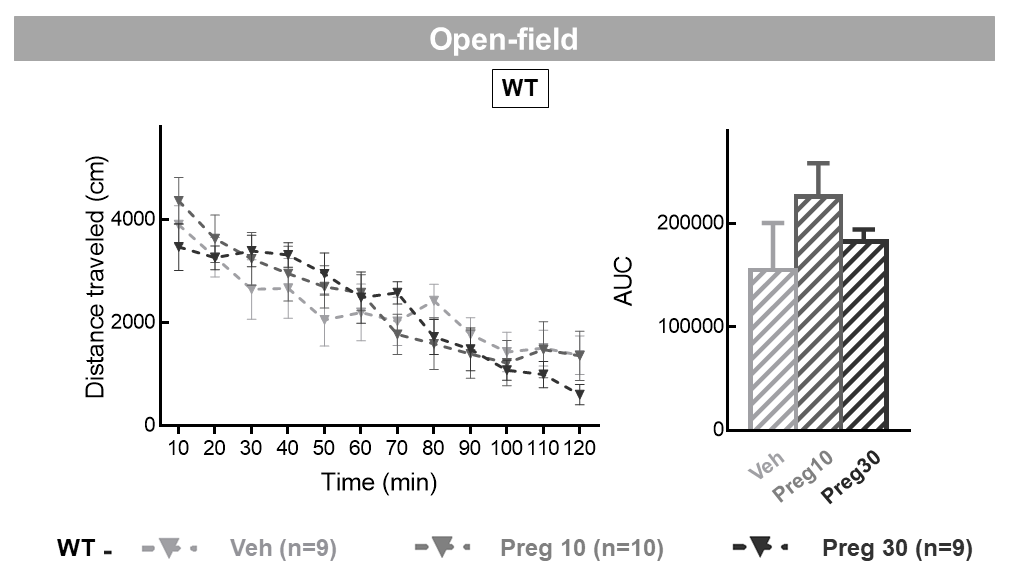

Supplement: Supplementary Figure S2 — Pharmacological evaluation of pregabalin effects on locomotor activity using the open-field test in wild-type (WT) female mice. Evaluation of spontaneous locomotor activity using the open-field test. Locomotion was evaluated using the total distance traveled ± SEM for the overall 120 min session. For AUC, statistical analysis was performed using one-way ANOVA followed by post-hoc Tukey's test; NS. [file Image_2.TIF]
